# Effect of *Trichoderma* Bioactive Metabolite Treatments on the Production, Quality, and Protein Profile of Strawberry Fruits

**DOI:** 10.1021/acs.jafc.0c01438

**Published:** 2020-05-19

**Authors:** Nadia Lombardi, Anna Maria Salzano, Antonio Dario Troise, Andrea Scaloni, Paola Vitaglione, Francesco Vinale, Roberta Marra, Simonetta Caira, Matteo Lorito, Giada d’Errico, Stefania Lanzuise, Sheridan Lois Woo

**Affiliations:** † Department of Agricultural Sciences, University of Naples Federico II, 80055 Portici, Naples, Italy; ‡ Proteomics & Mass Spectrometry Laboratory, ISPAAM, National Research Council, 80131 Naples, Italy; § Department of Veterinary Medicine and Animal Productions, University of Naples Federico II, 80138 Naples, Italy; ∥ Institute for Sustainable Plant Protection, National Research Council, 80055 Portici, Naples, Italy; ⊥ Task Force on Microbiome Studies, University of Naples Federico II, 80131 Naples, Italy; # Department of Pharmacy, University of Naples Federico II, 80131 Naples, Italy

**Keywords:** Fragaria x ananassa, Trichoderma, bioactive metabolites, antioxidant, polyphenols, anthocyanins, proteomics

## Abstract

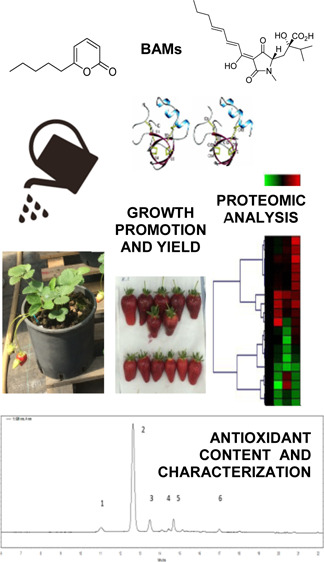

Fungi of the genus *Trichoderma* produce secondary metabolites having several biological activities that affect plant metabolism. We examined the effect of three *Trichoderma* bioactive metabolites (BAMs), namely, 6-pentyl-α-pyrone (6PP), harzianic acid (HA), and hydrophobin 1 (HYTLO1), on yield, fruit quality, and protein representation of strawberry plants. In particular, 6PP and HA increased the plant yield and number of fruits, when compared to control, while HYTLO1 promoted the growth of the roots and increased the total soluble solids content up to 19% and the accumulation of ascorbic acid and cyanidin 3-*O*-glucoside in red ripened fruits. Proteomic analysis showed that BAMs influenced the representation of proteins associated with the protein metabolism, response to stress/external stimuli, vesicle trafficking, carbon/energy, and secondary metabolism. Results suggest that the application of *Trichoderma* BAMs affects strawberry plant productivity and fruit quality and integrate previous observations on deregulated molecular processes in roots and leaves of *Trichoderma*-treated plants with original data on fruits.

## Introduction

Strawberry (*Fragaria x ananassa*) fruits, consumed as fresh and prepared food products, have been appreciated since ancient times for their taste, attractive visual, and nutritional properties.^1,2^ Because of its economic and commercial importance, the strawberry is highly studied, as demonstrated by the number of dedicated agronomic, genomic, and nutritional investigations.^3−8^ The consumer’s preference for strawberry is determined by different quantitative and qualitative parameters, such as fruit size and color, including health properties that are associated with the high content of antioxidant compounds, that is, vitamin C, proanthocyanidins, and anthocyanins (cyanidin and pelargonidin derivatives).^6,9,10^ Fruit quality, defined in terms of taste or other sensory and nutritional parameters, is strictly dependent on the content of soluble sugars.^11^

Studies have indicated that the application of beneficial microorganisms in crop production systems can have positive effects on plant disease control and plant growth, yield, and food quality.^12−15^ Fungi belonging to the genus *Trichoderma* are successful biocontrol agents (BCAs), marketed for their biopesticide and plant biostimulant activities. *Trichoderma* spp. are active ingredients in over 200 commercial products marketed worldwide.^16^ These bioformulations are successfully used as alternatives to the chemicals extensively used in conventional farming systems. The recent changes in European policy clearly promote more sustainable agriculture practices to reduce the risks to human and environmental health [see Regulation (EC) no. 1107/2009 of the European Parliament and of the Council of 21 October 2009, Concerning the Placing of Plant Protection Products on the Market and Repealing Council Directives 79/117/EEC and 91/414/EEC Latest consolidated version: 15/07/2019].^17^ Furthermore, these *Trichoderma* biological products are permitted in organic farming and recommended for use in the cultivation of agrifood or medicinal plants.

The majority of biological products applied in agriculture contain the dormant conservation structures of the living microbes, i.e., the spores of fungi, which represent the biological equivalent to the active substance found in synthetic chemical pesticides. Although microbial-based products are efficient, they have numerous disadvantages due to: (i) their limited shelf life and requirements for conservation in optimal conditions in order to maintain viability; (ii) constraints in the field due to crop, geographical, and meteorological regimes; and (iii) limited efficiency against some pathogens or short longevity in adverse environments.^18−20^

It has been well documented that the potential broad-spectrum antagonistic activity of fungal BCAs against various soil-borne phytopathogens and their ability to promote plant growth is attributed to the production of secondary metabolites.^21,22^ In particular, many *Trichoderma* strains are producers of bioactive metabolites (BAMs) that have growth-promoting and/or antimicrobial activities when applied to plants.^22−24^ Fravel reported that purified secondary metabolites from *Trichoderma* spp. could be potentially effective in controlling bacterial infections, exhibiting an antibiotic activity that was more rapid than that noted with the application of the living organism under field conditions.^25^ Similarly, further studies demonstrated that applications of *Trichoderma* bioactive compounds to different growing plants showed equivalent effects to those observed with the direct application of the metabolite producing strains but without the disadvantages of using the living microorganism in the agricultural system.^12,21,22,24^ Recently, Marra and co-workers observed that the application of different BAMs to soybean not only stimulated the plant growth but also increased the nutritional properties in the harvested seed.^14^ Similarly, Pascale and colleagues showed a positive correlation between the application of selected *Trichoderma* BAMs to grapevines and the increase of polyphenol content and antioxidant activity in the corresponding fruits.^12^ Among *Trichoderma* BAMs, hydrophobin HYTLO1, which has multiple roles and effects on treated plants,^28^ was able to trigger a nicotinic acid adenine dinucleotide phosphate-mediated Ca^2+^ signaling pathway in *Lotus japonicus*, highlighting a possible mechanism underlying its action.^29^

In the last decade, proteomic and metabolomic approaches have been largely used to describe the physiological changes occurring during the development, ripening, and post-harvest conservation of various fruits,^30−34^ including strawberry.^35−37^ Information is also available on the metabolic pathways and molecular processes involved in plant responses to treatments with the living *Trichoderma* fungus.^38^ In particular, proteomic and transcriptomic studies were performed on plant root, leaf tissues, or seedlings from maize, cucumber, tomato, bean, or grapevine treated with *Trichoderma harzianum* or *Trichoderma virens* to determine the quantitative changes in proteins/genes related to specific signaling cascades, defense response, redox stress, and carbon/energy metabolism pathways.^27,39−45^ A preliminary study has indicated differential gene/enzymatic activities of tomato fruits from plants treated with *T. harzianum*,^46^ but to date, no investigations have been specifically performed to evaluate the changes in the molecular and metabolic processes in fruits following treatments of plants with the compounds derived from the above-mentioned beneficial microorganisms.

This study investigated the effect of three BAMs from *Trichoderma*, that is, harzianic acid (HA), 6-pentyl-α-pyrone (6PP), and the hydrophobin (HYTLO1), on strawberry plants by evaluating the quantitative agronomic plant characteristics and the qualitative nutritional properties of the corresponding fruits, related to antioxidant compounds producing healthy effects to consumers.^6,47−50^ Differential proteomic analysis allowed the determination of the variable proteins related to the biosynthesis of these beneficial compounds produced by the fruits following BAM treatments. We used a broad spectrum identification of the metabolic pathways and molecular processes, which may be likely associated with the quality of strawberry fruits.

## Materials and Methods

### Chemicals

HA produced by *T. harzianum* strain M10 and HYTLO1 produced by *Trichoderma longibrachiatum* strain MK1 were extracted and purified from fungal culture filtrates as previously described by Vinale et al.^51^ (HA) and Ruocco et al.^28^ (HYTLO1). The 6PP metabolite was purchased from Sigma-Aldrich (St. Louis, MO, USA). Water, acetonitrile, methanol, and formic acid (FA) were of liquid chromatography–mass spectrometry (LC-MS) grade and purchased from Merck (Darmstadt, Germany) and Sigma-Aldrich. All reagents for proteomic analysis were from Sigma-Aldrich unless otherwise indicated.

### Plant Treatments and Fruit Processing

Young, uniform size fresh runner plants of *Fragaria x ananassa* cv. Sabrina were transplanted on October 2016 to pots (25 cm diameter, one plant per pot), containing sterile soil (Floragard potting soil, Oldenburg, Germany) placed on a raised bed, and grown in a protected greenhouse located at the Department of Agricultural Sciences of the University of Naples Federico II, Portici (Naples, Italy). The experimental design was a complete randomized block, containing four treatments: a water control (CTRL) and three *Trichoderma* metabolites (HA, HYTLO1, and 6PP), with two biological replicates per treatment, containing 10 plants in each replicate. HYTO1 was dissolved in 70% ethanol, HA and 6PP were dissolved in ethyl acetate (successively evaporated), and each compound was prepared to the final concentration of 10^–6^ M with distilled water. Metabolite solutions were applied by root dip (15 min soaking) at the time of transplant, then at monthly intervals by root watering (25 mL per plant), up to 7 days before the first harvest of the fruits.

When red ripened fruits were observed on the plants, the fruits were harvested once per week from April to June 2017 to determine the number and fresh weight of collected fruits per plant, plus the total soluble solids (TSSs) content. At the end of June 2017, after completion of fruit harvest, strawberry plants were removed from the pots; roots were rinsed with water to remove soil and measured, then plants were air-dried in a ventilated oven at 65 °C for 72 h until achieving a constant weight. For each treatment, total fruit yield (TY), number of fruits/plant (NF), root length (RL), root fresh weight (RFW), and root dry weight (RDW) were assessed.

Collected fruits were frozen in liquid nitrogen, then stored at −80 °C until analyzed. For the chemical analyses, strawberry fruits were freeze-dried; dried samples were pulverized, homogenized by using a knife mill Grindomix 200 (Retsch, Haan, Germany), and then stored in a desiccator in the dark at room temperature. Powdered strawberry samples were combined according to treatment and subjected to chemical analyses. For the proteomic analysis, collected fruits were frozen in liquid nitrogen and then stored at −80 °C until analyzed. Frozen strawberries (nine fruits from three plants) were pooled according to treatment, pulverized using a laboratory blender, ground in a mortar in the presence of abundant liquid N_2_, and finally lyophilized before further processing.

### Chemical Analysis of the Fruits

#### TSSs Content

TSSs content was determined at the time of each harvest by using a Brix hand refractometer (RF.5520 Euromex, Arnhem, The Netherlands), and values were reported as degrees Brix (°Brix). Ten fruits were used for each treatment, whereby the fruits were cut into two parts, and each half was squeezed to obtain a measurement of the refractive index of the juice extract.

### TAC, Total Phenolic and Ascorbic Acid Content in Fruits

Total antioxidant capacity (TAC) of hydroalcoholic extracts from strawberry fruits was monitored through the α,α-diphenyl-β-picrylhydrazyl (DPPH) free radical assay according to the procedure described by Sharma and Bhat.^52^ In brief, hydroalcoholic suspensions (10 mg/mL in methanol/water/FA, 70/29/1, v/v/v) were homogenized and centrifuged at 2500 × *g* for 10 min, at 4 °C; 0.2 mL of hydroalcoholic extracts was added to 0.9 mL of DPPH (Sigma-Aldrich, adjusted absorbance 0.9 ± 0.02) dissolved in methanol (0.4 mg/mL) and incubated at 20 °C (10 min). The scavenging capacity was measured by the absorbance at 517 nm with a T92+ UV double beam spectrophotometer (PG Instruments, Leicester, UK). Methanol/water/FA solution was used to evaluate the percentage of inhibition. Measurements were calibrated in the range 10–120 μM by using (±)-6-hydroxy-2,5,7,8-tetramethylchromane-2-carboxylic acid (trolox) (Sigma-Aldrich) as a standard; TAC was reported as μmol equivalent of trolox per gram of fruit dry matter.

The Folin–Ciocalteu method was used to evaluate sample total phenolic content (TPC) according to Singleton.^53^ Water (0.5 mL) and 125 μL of a Folin–Ciocalteu solution (Sigma-Aldrich) were added to the above-mentioned hydroalcoholic suspensions (0.1 mL) and incubated for 6 min at room temperature. Then, 1.25 mL of 0.70 M sodium carbonate was added, vortexed, and further incubated for 90 min. Sample absorbance was measured at 760 nm. Measurements were calibrated in the range 0.020–0.150 mg/mL by using gallic acid (Sigma-Aldrich) as a standard.

For ascorbic acid quantitation, powdered strawberry samples (0.5 g) were suspended in a 5 mL solution of 0.3 M metaphosphoric acid and 1.3 M acetic acid and centrifuged at 2500 × *g* for 10 min at 4 °C; supernatants were titrated using 25% (w/v) 2,6-dichloroindophenol and 21% (w/v) sodium hydrogen carbonate. Ascorbic acid was used as a reference standard for calibration curves.^54^

### Characterization of Individual Anthocyanins

Anthocyanin extraction was performed according to Holzwarth and co-workers,^55^ with minor modifications. Dried strawberry samples (50 mg) were dissolved in FA/methanol solution (3 mL, 5/95, v/v), sonicated (10 min, at 40 °C), and incubated at 40 °C in agitation (20 min). Subsequently, the samples were centrifuged at 2500 × *g*, for 10 min, at 4 °C, and the supernatants were collected separately (1 mL aliquots) and dried in a Speed-vac (Savant, Thermo-Fisher, Bremen, Germany) at 40 °C. The precipitate was suspended in 0.3 mL of aqueous FA (5% v/v), and then, the samples were subjected to liquid chromatography diode array detector (LC-DAD) analysis to determine the single anthocyanin contents by using a LC10AD binary system, SPD-M10A DAD, and controller SCL 10A (Shimadzu, Kyoto, Japan) connected to a Series 200 autosampler (PerkinElmer, Billerica, MA). Anthocyanins separation was achieved on a Kinetex XB-C18 column (150 × 4.6 mm, 5 μm, 100 Å, Phenomenex, Torrance, CA, USA) equipped with a security guard column of the same stationary phase at a flow rate of 0.8 mL/min. Analytes were eluted with a binary mixture of mobile phase A (5% FA in water) and B (5% FA in methanol) with the following gradient (min/% B): (0/20), (3/20), (15/55), (18/55), (22/90), (25/90). Compounds were monitored at 520 nm and assigned by *on-line* (splitting ratio of 1/10) MS analysis with an API2000 triple quadrupole tandem mass spectrometer (ABSciex, Carlsbad, CA). Positive electrospray ionization was used, while ion source parameters were as follows: spray voltage 5.5 kV; capillary temperature 300 °C; and dwell time 100 ms. Tentative identification of anthocyanins was achieved according to Määttä-Riihinen et al.^56^ using mass transitions provided in parentheses in the multiple reaction monitoring (MRM) mode: cyanidin 3-*O*-glucoside (*m*/*z* 449 → 287), pelargonidin 3-*O*-glucoside (*m*/*z* 433 → 271), pelargonidin 3-*O*-rutinoside (*m*/*z* 579 → 271), pelargonidin 3-*O*-malonyl-glucoside (*m*/*z* 519 → 271), pelargonidin 3-*O*-acetyl-glucoside (*m*/*z* 475 → 271), and cyanidin derivative (*m*/*z* 449 → 287).

Each sample was extracted and injected twice (*n* = 4), and results were reported as μg/g of the sample. Metabolite calibration curves were built in the range 0.1–50 μg/mL. Individual anthocyanins and anthocyanidins (aglycone form) lacking the corresponding standard compound were quantified using calibration curves of pelargonidin 3-*O*-glucoside and cyanidin (Extrasynthése, Lyon, France), respectively, after proper correction according to Chandra and colleagues.^57^

### Fruit Protein Extraction, Digestion, and Peptide Fractionation

Proteins were extracted from powdered fruit samples according to Li et al.^37^ with minor modifications regarding phenol extraction and precipitation with methanolic ammonium acetate. Briefly, 1 g of lyophilized sample was mixed with 0.01 g of polyvinylpyrrolidone (Sigma-Aldrich) and then resuspended in 10 ml of 0.7 M sucrose, 0.1 M KCl, 0.5 M Tris–HCl, 50 mM EDTA, 40 mM DTT, pH 8.5, supplemented with a protease inhibitor mix for plant tissues (Sigma-Aldrich). Each suspension was homogenized with an Ultra-Turrax device (IKA, Werke GmbH Germany) at 6000 rpm for 1 min. Tris-buffered phenol (pH 8.0) (Sigma-Aldrich) was added to each sample (1:1, v/v), and phase separation was obtained by centrifugation (10 000 × *g*, for 15 min, at 4 °C). The extraction was performed twice, and collected phenolic phases were precipitated with 5 vol of ice-cold 0.1 M methanolic ammonium acetate at −20 °C overnight. Samples were centrifuged (8000 × *g*, for 10 min, at 4 °C), then the protein pellets obtained were washed twice with ice-cold methanol and once with cold acetone containing 20 mM DTT and then air-dried. Pellets (5 mg) were dissolved in 250 μL of 7 M urea, 2 M thiourea, 50 mM triethylammonium bicarbonate (TEAB), 2% SDS, 10 mM DTT, pH 8.5, containing the protease inhibitor mix (Sigma-Aldrich), vortexed, and incubated at 30 °C, for 1 h, under gentle shaking. Samples were then centrifuged (12 000 × *g*, for 5 min, at 4 °C), the supernatants were independently recovered, and protein concentration was determined using the Bio-Rad Protein Assay (Bio-Rad Hercules, CA, USA) according to the manufacturer’s instructions.

Relative quantification of individual proteins was obtained by performing a labeling-based experiment using the TMT10plex Isobaric Label reagent kit (Thermo-Fisher Scientific, USA). For each sample, 100 μg of the protein extract was adjusted to 100 μL with 100 mM TEAB and treated as described in the manufacturer’s instructions to obtain the corresponding tryptic digest. Peptides from each sample were chemically labeled with one of the reagents from the TMT10plex kit according to the labeling scheme: CTRL-TMT10-126, HA-TMT10-128C, 6PP-TMT10-129N, and HYTLO1-TMT10-129C. Labeling proceeded for 1 h, and then, the reaction was quenched by the addition of 8 μL of 5% w/v hydroxylamine for 15 min. Labeled peptide mixtures were mixed at an equal molar ratio (1:1:1:1), dried, resuspended in 0.1% trifluoroacetic acid, and fractionated with the Pierce high pH reversed-phase peptide fractionation kit (Thermo-Fisher Scientific) according to the manufacturer’s instructions. Eight fractions of TMT-labeled peptides were collected, dried, and finally reconstituted in 0.1% (v/v) aqueous FA before mass spectrometric analysis.

### NanoLC-ESI-Q-Orbitrap MS/MS Analysis of Fruit Protein Digests

Analysis of labeled peptide fractions was carried out through nanoLC-ESI-Q-Orbitrap-MS/MS experiments performed with a platform consisting of an UltiMate 3000 HPLC RSLC nanosystem (Dionex, USA) interfaced to a Q-ExactivePlus Hybrid Quadrupole-Orbitrap mass spectrometer through a Nanoflex ion source (Thermo-Fisher Scientific). Peptides were injected on an Acclaim PepMapTM RSLC C18 column (150 mm × 75 μm ID, 2 μm particles, 100 Å pore size) (Thermo-Fisher Scientific) and eluted with a gradient of solvent B (80% acetonitrile, 20% H_2_O plus 0.1% FA) in solvent A (100% H_2_O plus 0.1% FA) at a flow rate of 300 nL/min. The gradient was as follows: from 5 to 60% of solvent B over 125 min and from 60 to 95% solvent B over 1 min. The column was washed after each chromatographic run and re-equilibrated at 5% solvent B for 20 min before the subsequent analysis. Data-dependent acquisition was selected as the operating mode for the mass spectrometer. Full scans were acquired in the *m*/*z* range 375–1500 at a resolution of 70,000. MS/MS analyses were performed on the 10 most abundant ions from the preceding full scan. A dynamic exclusion duration of 30 s was used. MS/MS spectra were acquired with a resolution of 17,500 in the scan *m*/*z* range 110–2000. Isolation window and normalized collision energy were set at *m*/*z* 1.2 and 32%, respectively. Automatic gain control target and maximum ion target were set at 100,000 and 120 ms, respectively.

### Bioinformatics—Protein Identification, Quantification, and Functional Analysis

Raw mass spectrometric data were analyzed by using Proteome Discoverer (PD) v2.1 software (Thermo Scientific) to generate protein identification and relative quantification results. Mascot algorithm v. 2.6 (Matrix Science, UK) was used within PD software, together with a plant protein database retrieved from NCBI (Viridiplantae, 6216064 protein sequences, 12/2018), also including the most common protein contaminants. Carbamidomethylation of Cys and TMT modification of lysine and peptide N-terminus were set as fixed modifications. Oxidation of Met, deamidation of Asn/Gln, and pyroglutamate cyclization from Gln were selected as variable modifications. Mass tolerance values were 10 ppm for parent ions and 0.02 Da for fragments. Maximum missed cleavages for trypsin were set to 2. Protein candidates were assigned if at least two peptides were confidently assigned with an individual Mascot score ≥ 30. Results were filtered to keep only high confidence identification results (corresponding to a false discovery rate of 1%). For relative protein quantification, PD software calculated abundance ratios between experimental samples from the ratios of TMT reporter ion intensities in the MS/MS spectra. Proteomic data were deposited to the ProteomeXchange consortium^58^ within the PRIDE partner repository with the dataset identifier PXD016951.

Identified proteins were subjected to sequence homology search using command line BLAST applications against the *Arabidopsis thaliana* protein sequence database (TAIR 10) retrieved from The Arabidopsis Information Resource repository. Functional analysis of differentially represented proteins (DRPs) and corresponding graphical representations were performed as previously described.^33^

### Statistical Analysis

Statistical analysis was conducted on all measured parameters: the biometric parameters (TY, NF, RL, RFW, and RDW), TAC, TPC, as well as content of TSSs, ascorbic acid, total anthocyanins, and individual anthocyanins were evaluated by one-way ANOVA using SPSS software (v.15.0 IBM, Armonk, NY). Significant differences among treatments were determined by using S–N–K (Student–Newman–Keuls) and Fisher’s least significant difference *post hoc* tests at the 0.05 level of significance.

## Results

### Strawberry Yield and Plant Growth

All three *Trichoderma* metabolites had an effect on strawberry plant growth and productivity, but the results were not equivalent among the compounds, and actually in many cases the outcomes were contrasting for the measured biometric parameters. Treatments with HA and 6PP significantly enhanced (both +24%) the TY (*P* < 0.05), as compared to control (CTRL), whereas HYTLO1 had an opposite effect, significantly reducing TY (−22%) (Table 1). Similarly, both applications of HA and 6PP increased the number of fruits per plant (NF), but the influence of 6PP on the productivity was more significant (+36% vs CTRL), in comparison to HA (+14%), while a detrimental effect was observed in the case of HYTLO1 (−17%). On the other hand, treatments with the *Trichoderma* compounds HA or 6PP did not significantly affect the strawberry plant growth in relation to root development (Table 1). Only HYTLO1 determined in treated plants an increase in RL (+15% compared to CTRL), RFW (+15%), and RDW (+19%) (Table 1).

**Table 1:** Effects of Different *Trichoderma* BAMs (HA, 6PP, and HYTLO1) on the Growth and Productivity of Strawberry Plants under Greenhouse Conditions^a^

	TY (g plant^–1^)		number of fruits plant^–1^ (NF)		RL (cm plant^–1^)		RFW (g plant^–1^)		RDW (g plant^–1^)	
treatment	mean ± SD	%	mean ± SD	%	mean ± SD	%	mean ± SD	%	mean ± SD	%
CTRL	125.4 ± 21.8 b		6.4 ± 1.2 ab		22.0 ± 1.9 a		62.9 ± 5.4 ab		13.5 ± 1.1 bc	
HA	155.9 ± 16.4 c	24	7.3 ± 1.9 bc	14	23.9 ± 2.3 ab	9	64.4 ± 7.3 ab	2	13.2 ± 2.1 ab	–2
6PP	155.2 ± 18.0 c	24	8.7 ± 1.2 d	36	23.3 ± 3.4 ab	6	61.7 ± 9.6 a	–2	11.6 ± 2.2 a	–14
HYTLO1	97.6 ± 21.4 a	–22	5.3 ± 1.6 a	–17	25.2 ± 1.9 b	15	72.1 ± 8.2 d	15	16.1 ± 1.8 c	19

a Treatments were applied at the time of transplant (root dip) and monthly by irrigation. Data represent the mean value of 10 biological replicates ± SD. Different letters in a single column indicate statistically significant differences for *P* < 0.05. Increments or decrements compared to control (CTRL) are shown in percent (%).

### Strawberry Fruit Nutritional Characteristics

Analysis of strawberry fruits demonstrated that the applications of the *Trichoderma* metabolites to the growing plants had an effect on nutritional characteristics of the products. HA and HYTLO1 treatments resulted in a significant increase of the TSSs content (+8 and +19%, respectively, compared to the water control) (Table 2). Only HYTLO1 had a positive, although not significant, effect on the ascorbic acid content (+9%) (Table 2).

**Table 2:** Effects of the Application of Different *Trichoderma* BAMs (HA, 6PP, and HYTLO1) on TSSs and the Antioxidant Properties of Strawberry Fruits^a^

	TSSs (°Brix)		antioxidant capacity [μmol equiv Trolox g^–1^]		total polyphenols [mg g^–1^]		ascorbic acid [mg 100 g^–1^]		total anthocyanins [μg g^–1^]	
treatment	mean ± SD	%	mean ± SD	%	mean ± SD	%	mean ± SD	%	mean ± SD	%
CTRL	9.9 ± 1.0 a		54.0 ± 10.7 bc		10.9 ± 0.1 a		116.8 ± 12.6 bc		809.0 ± 13.0 ab	
HA	10.7 ± 1.6 b	8	46.6 ± 1.8 ab	–14	9.8 ± 0.9 a	–10	111.5 ± 5.0 b	–5	572.9 ± 14.7 bc	–29
6PP	9.7 ± 1.2 a	–2	40.8 ± 1.9 a	–24	8.7 ± 0.3 a	–20	90.0 ± 0.0 a	–23	671.9 ± 16.4 bc	–17
HYTLO1	11.8 ± 1.3 c	19	51.9 ± 6.1 abc	–4	10.0 ± 2.0 a	–9	127.9 ± 29.9 c	9	691.6 ± 13.2 bc	–15

a Treatments were applied at the time of transplant (root dip) and monthly by irrigation. Data represent the mean value of eight biological replicates ± SD. Different letters in a single column indicate statistically significant differences for *P* < 0.05. Increments or decrements compared to control (CTRL) are reported as %.

**Figure 1:**
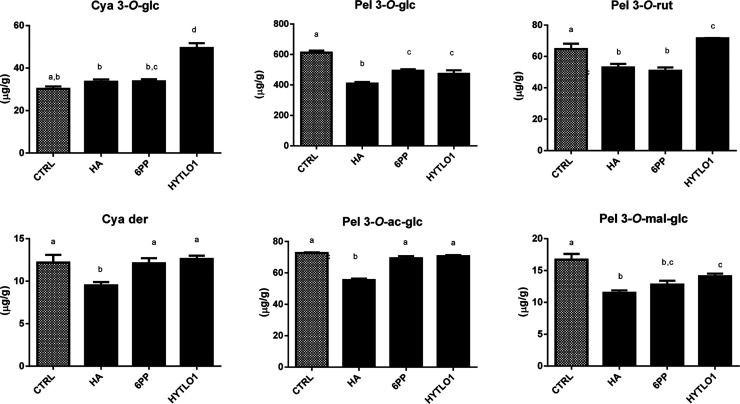
Concentration of individual anthocyanins in strawberry fruits produced by plants subjected to the treatment with different *Trichoderma* BAMs (HA, 6PP, and HYTLO1), as compared to control (CTRL). Results on cyanidin 3-*O*-glucoside (cya 3-*O*-glc), pelargonidin 3-*O*-glucoside (pel 3-*O*-glc), pelargonidin 3-*O*-rutinoside (pel 3-*O*-rut), pelargonidin 3-*O*-malonyl-glucoside (pel 3-*O*-mal-glc), pelargonidin 3-*O*-acetyl-glucoside (pel 3-*O*-ac-glc), and cyanidin derivative (cya der) are shown. Data were reported as μg/g sample and represent the mean value of eight biological replicates ± standard deviation (SD). Different letters in a single column indicate statistically significant differences for P < 0.05 according to One-way ANOVA with post hoc Tukey HSD Test.

To further analyze the effect of *Trichoderma* metabolites on the accumulation of the anthocyanins, a quali-quantitative characterization of individual compounds in the strawberry fruits produced by plants subjected to the treatment with different BAMs was carried out. Tentative identification was achieved in MRM mode following the chemical behavior of individual benzopyrylium and flavylium ions, as conducted in previous investigations.^6,7,11^ Compounds generating a fragment signal at *m*/*z* 287 were assigned to members of the cyanidin family, while those producing a signal at *m*/*z* 271 were assigned to the pelargonidin family (Table S1). Combined with tentative mass spectrometric identification, LC–DAD-assisted quantitative measurements confirmed a different accumulation of six individual anthocyanins in the fruits according to the BAM treatment to the plant (Figure 1). In particular, an overall decrease (*P* < 0.05) in the content of the most abundant anthocyanin, pel 3-*O*-glc, and in pel 3-*O*-mal-glc was observed for all the three treatments (Figure 1). A net decrease in the concentration of five out of the six anthocyanins was noted following the application of HA, that is, −33% pel 3-*O*-glc versus CTRL, −31% pel 3-*O*-mal-glc, −24% pel 3-*O*-ac-glc (Figure 1). Conversely, the application of HYTLO1 increased significantly the content of cya 3-*O*-glc and pel 3-*O*-rut (+63 and +11%, respectively, compared to control). Table S1 and Figure S1 report MS transitions used for the anthocyanin profile characterization of strawberry fruits from BAM-treated plants, along with a representative chromatographic profile.

### Proteomic Analysis of Strawberry Fruits

**Figure 2:**
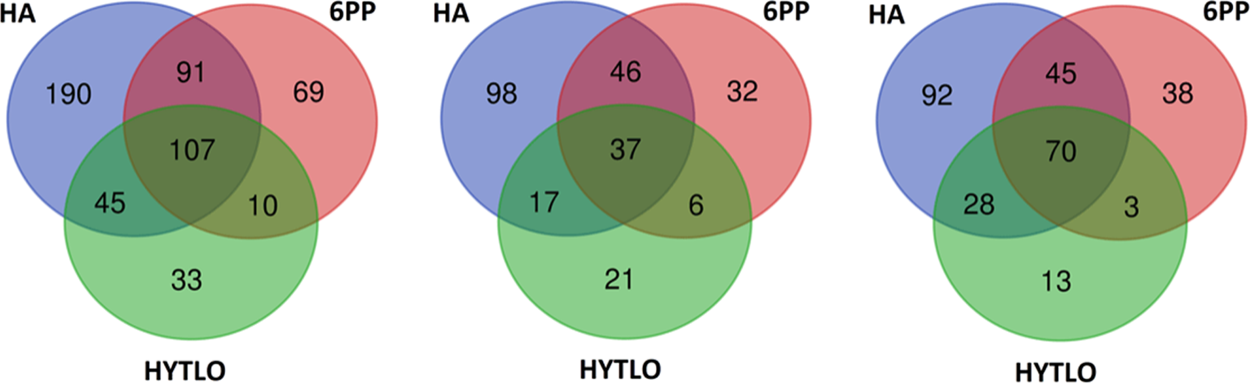
Venn diagram showing DRPs present in strawberry fruits produced by plants subjected to the treatment with different *Trichoderma* BAMs (HA, 6PP, and HYTLO1), as compared to control. Diagrams refer to all DRPs (left), those down-represented (middle), and over-represented (right), respectively.

A TMT-based quantitative proteomic analysis was conducted to determine the effects of *Trichoderma* BAMs on protein effectors and metabolic pathways in strawberry fruits obtained from treated plants. This analysis allowed the identification of 3294 fruit proteins and, among them, measured the relative amount of 3014 compounds, which were in turn associated, respectively, with 3262 and 2982 nonredundant *A. thaliana* sequence entries present in the TAIR 10 database, plus 32 extra ones not having a sequence homologue therein (data repository PRIDE PXD016951). In the strawberry fruits, 545 components were identified as DRPs produced after treatments with the *Trichoderma* compounds when abundance fold changes ≤0.66 or ≥1.50 (*P* ≤ 0.05), and accession redundancy were considered (Table S2). These DPRs were associated with 528 nonredundant *A. thaliana* sequence entries in the TAIR 10 database, plus 17 extra ones not having a sequence homologue therein. The HA, 6PP, and HYTLO1 treatments determined in strawberry fruits the accumulation of 433, 277, and 195 DRPs, respectively, whose unique and shared species are reported in a dedicated Venn diagram (Figure 2). Furthermore, 257 down- and 289 over-represented protein species were noted. Hierarchical clustering of abundance ratios and distribution of DRPs among treatments showed that HA determined the most represented variations compared to control in both over- (235) and down-represented (198) proteins, followed by 6PP (determining 156 and 121 over- and down-represented proteins, respectively) and HYTLO1 (determining 114 and 81 over- and down-represented proteins, respectively) (Figure 2).

**Figure 3:**
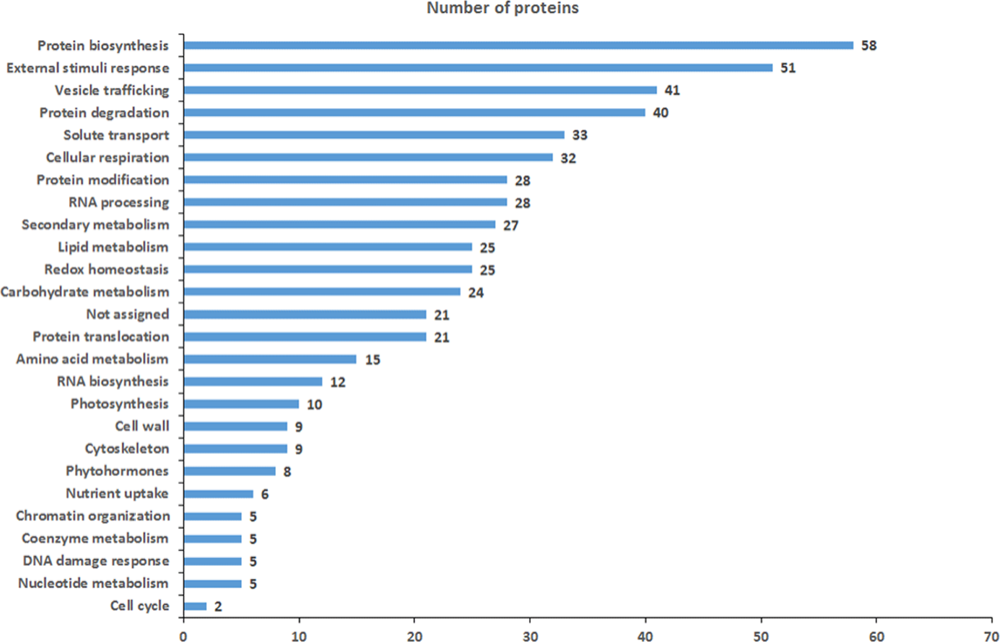
Functional distribution of DRPs in strawberry fruits produced by plants subjected to the treatment with different *Trichoderma* BAMs (HA, 6PP, and HYTLO1), as compared to control. Identified protein species were initially assigned with Mercator software^88^ (Table S3), followed by a functional group cataloguing including information from the Bevan classification^89^ and recent literature data.

The analysis of the functional assignment of DRPs was at first obtained with Mercator software, which was integrated with information from the Bevan classification and recent literature data (Figure 3 and Table S3). Results indicated that only 21 proteins were not allocated to known functional and/or ontology groups using this procedure. Most represented functional categories of DRPs highlighted significant molecular processes and metabolic pathways resulting to be affected by treatment with *Trichoderma* BAMs. These included the following: (i) protein metabolism (molecules involved in protein biosynthesis/translocation/degradation: 21.8%); (ii) stress response (proteins related to redox homeostasis, external stimuli response, and protein modification: 19.1%); (iii) carbon and energy metabolism (enzymes associated with carbohydrate metabolism, energy, and photosynthesis: 12.1%); (iv) vesicle trafficking (7.5%); (v) RNA metabolism (molecules involved in RNA biosynthesis/processing: 7.3%); and (vi) secondary metabolism (enzymes involved in biosynthesis/degradation of secondary metabolites/phytohormones: 6.4%). No dissimilarity in the functional distributions was observed when DRPs from 6PP, HA, and HYTLO1 were independently evaluated (data not shown). Functional enrichment of DRPs for the biological process and molecular function (GO) and KEGG pathways established the participation of most of the strawberry fruit proteins in response to a spectrum of chemical stimuli, in binding to ions/small molecules, in redox processes, and in different catalytic activities, as well as in biosynthesis of secondary metabolites, protein processing, oxidative phosphorylation, and carbon metabolism (Table S4).

Figures 4 and S2–S13 show heat-map representations deriving from hierarchical grouping of quantity ratios of DPRs for each functional class. They describe the relative quantitative outline of strawberry DPRs as an outcome of the diverse *Trichoderma* BAM applications. In general, a coherent tendency of DRPs was clearly notable with the *Trichoderma* treatments to the strawberry plants versus the control, as well as among the three diverse treatments, where very few exceptions were detected.

**Figure 4:**
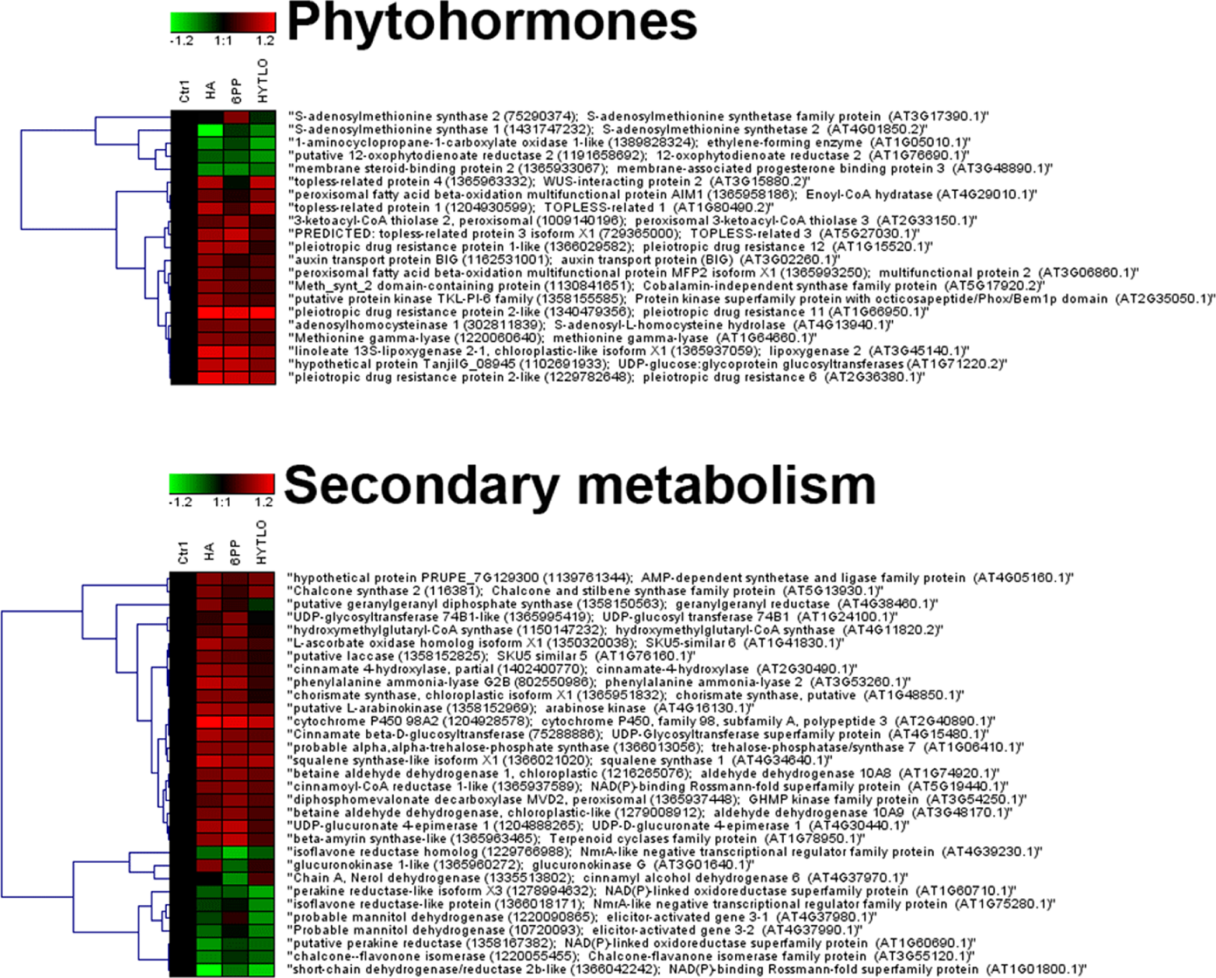
Heat-map representation and hierarchical clustering analysis of proteins related to phytohormone metabolism (upper panel) and secondary metabolism (lower panel), which were present in strawberry fruits produced by plants subjected to the treatment with different *Trichoderma* BAMs (HA, 6PP, and HYTLO1), as compared to control (CTRL). Shown are proteins presenting abundance fold changes ≥1.50 or ≤0.66 with respect to control (*P* ≤ 0.05). Data are reported as log_2_ transformed abundance ratio values. Hierarchical clustering analysis of DRPs was performed using Genesis 1.8.1 platform (Institute for Genomics and Bioinformatics, Graz University of Technology).

**Figure 5:**
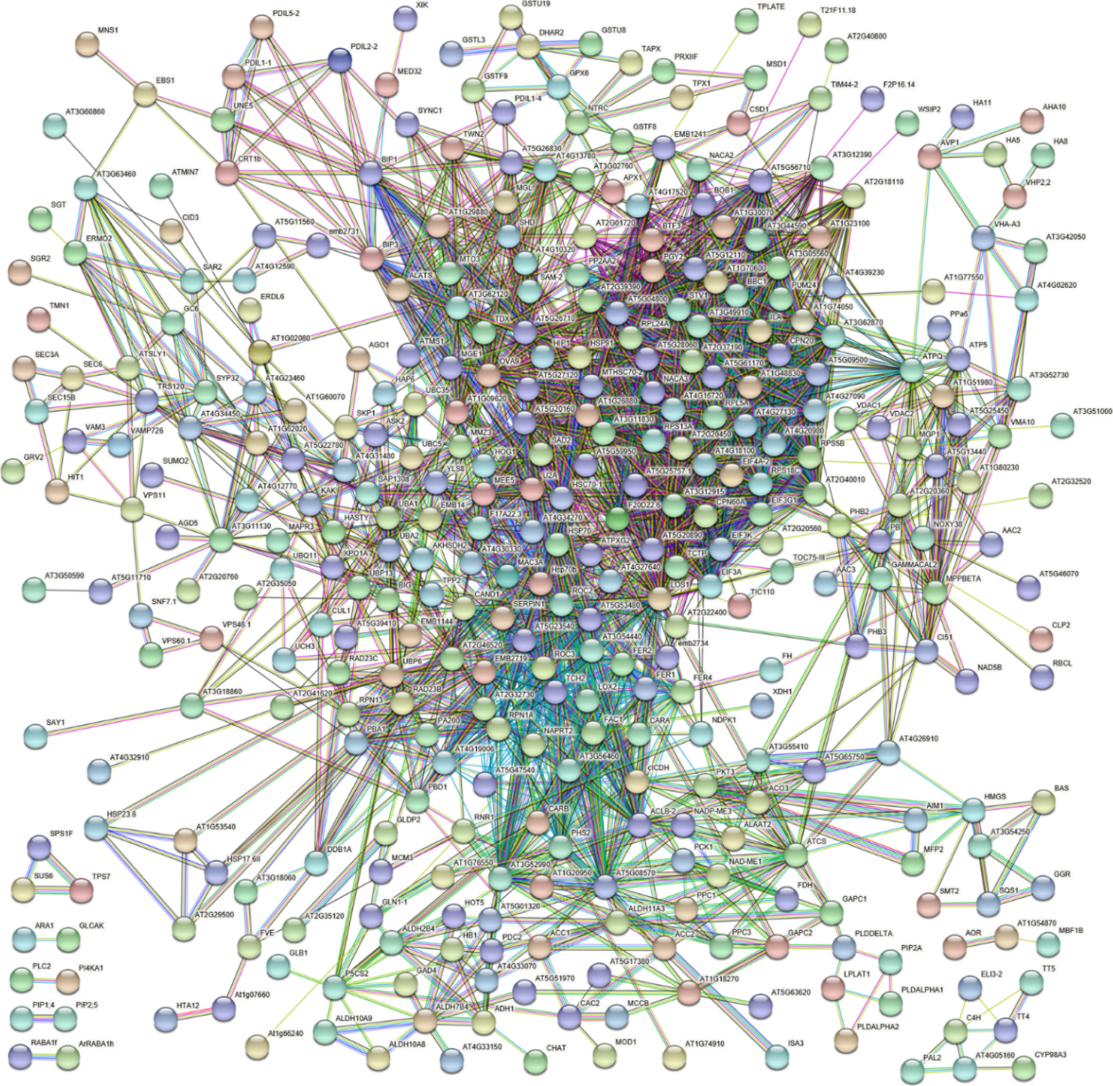
STRING analysis of DRPs present in strawberry fruits produced by plants subjected to the treatment with different *Trichoderma* BAMs (HA, 6PP, and HYTLO1), as compared to control. Functional protein associations were based on data recorded for *A. thaliana* protein homologues. Only high-confidence interactions (0.7) are shown. Protein codes are reported in Table S5.

When STRING software was used at high confidence (0.7) and based on *A. thaliana* protein homologues to predict an association map between strawberry DRPs, a central network connecting 354 components was observed (Figure 5 and Table S5). This predominant network involved 64.9% of ascertained DRPs, thus suggesting the possible, coordinated regulation of various molecular processes and metabolic pathways as a consequence of the treatment with *Trichoderma* BAMs. Most of the network knots were related to the HA treatment.

In conclusion, proteomic results provided evidence that the application of *Trichoderma* BAMs on strawberry plants regulates several metabolic, energetic, and signaling processes in the corresponding fruits, as well as a series of molecular mechanisms related to plant response to external stimuli/stresses.

## Discussion

This investigation evaluated the effects of diverse *Trichoderma* BAMs (6PP, HA, HYTLO1) applied to strawberry plants on productivity and quality of their corresponding fruits. Previous studies with these BAMs have demonstrated positive effects to diverse plant species important to agriculture, which include improved plant growth, disease pathogen control, and induction of resistance.^26,28^ To this end, the potential outcome on strawberry, a crop cultivated in small fruit production, was examined to ascertain if advantages observed on the plant after BAM treatments were also transferred to the edible fruit structures. Measurements of the biometric parameters of the plant, such as the root length, fresh and dry weight (RL, RFW, and RDW), plus total yield (TY), and number of fruits (NF), were evaluated and associated with the fruit quality parameters. These included the TSSs content, TAC, total phenolic content (TPC), and concentrations of ascorbic acid and total and individual anthocyanins. Results confirmed the ability of the selected *Trichoderma* metabolites to act as growth promotion agents; in particular, HA and 6PP treatments increased TY and NF values, compared to control (Table 1). This confirmed the auxin-like activity of the *Trichoderma* 6PP metabolite to stimulate plant growth, as previously described by Vinale et al.^22^ and Pascale et al.^12^ On the other hand, HYTLO1 showed no effects on the above ground structures while promoting the growth of the root system (in terms of RL, RFW, and RDW; Table 1). These results are in agreement with previous findings in HYTLO1-treated tomato plants, which demonstrated significant stimulation of the below ground structures.^28^

Proteomic results provided a rationale to the above-mentioned results based on the numerous, common over-represented proteins induced by HA, 6PP, and HYTLO1, which are involved in nutrient import and/or associated with the occurrence of essential chemicals in the plant cell environment (Table S4). In this context, noteworthy are various plasma membrane proteins and membrane transporters involved in the cell uptake of H_2_O and neutral (nucleotide, base, sugar-phosphate, vitamin, and organic) compounds,^59^ such as H^+^- and Ca^2+^-translocating ATPases coupling ion fluxes implicated in energy production of the plant,^38,60^ ABCG transporters involved in phytohormone and oxidant translocation, and H^+^-exporting pyrophospatases (Figure S2). The latter proteins/transporters have been previously reported to assist plant growth processes including auxin-based leaf/fruit growth, biomass increase, and improved fruit yield.^61,62^ On the other hand, sugar-phosphate transporters have already been described as partners of glycosyltransferases in the biosynthesis of oligo-/polysaccharides and glycoconjugates.^63^ In line with the observed growth-promoting effect of *Trichoderma* BAMs were also the reduced levels of chloroplast N-regulatory protein PII (GlnB) isoforms, which confirmed the already reported down-regulation of the corresponding genes in the presence of high concentrations of nitrogenous compounds^64^ (Figure S2). These proteins have been reported to modulate target enzymes depending upon the plant cell ADP/ATP and 2-oxoglutarate concentrations, thus regulating organism C/N balance through the dedicated metabolic processes.^65^ The highest quantitative changes of some of the above-mentioned plasma membrane proteins, membrane transporters, and GlnB isoforms detected in HA- and 6PP-treated plants (Figure S2), with respect to HYTLO1-treated counterparts, hypothetically provided a rationale to measured TY and NF values in these organisms.

Proteomics can also provide information on the processes that augment energetic stream and growth of various plant organs. In this context, the number of over-represented enzymes involved in carbohydrate (starch, sucrose, and nucleotide-sugar) anabolism/catabolism, glycolysis, the Krebs cycle, and alcoholic fermentation here detected in the fruits produced from the plants treated with the *Trichoderma* metabolites (Figure S3) was in agreement with the findings of earlier studies conducted on leaves and roots of tomato, grapevine, maize, and cucumber plants instead treated with living *Trichoderma* fungus-based formulations.^27,38,40,41,66^ Furthermore, particularly with the HA treatment, we noted an augmented level of the sucrose nonfermenting 4-like protein isoform X1, a kinase metabolic regulator of carbohydrate metabolism and polysaccharide biosynthesis that positively affects starch, sucrose, glucose, and fructose accumulation in plants.^67,68^ These proteomic results provided a metabolic rationale to the increased levels of soluble sugars noted in fruits from BAM-treated plants (Table 2), which justifies the shift toward energy production in order to sustain the observed increase in plant growth. In contrast, all protein elements of mitochondrial cytochrome C reductase and ATP synthase complexes assisting energy production were down-represented following the BAM treatments (Figure S3). This observation suggests a condition in which there is detrimental generation of reactive oxygen species (ROS) during oxidative phosphorylation that was partially hampered in fruits, in an attempt to control oxidant concentration fluxes in BAM-treated plants through other stimulatory processes (see below).^38^ Finally, the modulation of enzymes controlling citrate concentration in fruits obtained from the treated plants suggests that the fungal BAMs can also regulate the whole fruit’s acidity.^69^ Again, the more pronounced variations measured in HA- and 6PP-treated plants for some of the above-mentioned carbon/energy metabolism enzymes (Figure S4), with respect to HYTLO1-treated counterparts, were tentatively related to the highest values of TY and NF detected in these organisms.

As expected, the observed improvement in the growth and development in *Trichoderma* BAM-treated plants corresponded to the augmented levels of enzymes involved in processes promoting the biosynthesis of essential metabolites and in the fueling precursor elements for the production of structural proteins in growing fruit cells.^41^ This was particularly evident for proteins affecting the following: (i) biosynthesis of amino acids (13 in number); (ii) biosynthesis of amino acid-tRNAs (12 in number); (iii) biosynthesis of lipids (15 in number); (iv) cell cycle (4 in number); (v) protein translocation toward various cell districts (14 in number); (vi) polypeptide chain translation initiation/elongation activities (8 in number); and (vii) nucleotide metabolism (4 in number) (Figures S5, S7, and S9). This observation found a similar match in the number of over-represented constitutive proteins associated with changes in the cell wall (7 in number) and cytoskeleton (6 in number) (Figure S12). General quantitative trends observed for enzymes involved in lipid metabolism were also in line with augmented levels of fatty acids previously measured in soybean plants treated with the same HA, 6PP, and HYTLO1 metabolites (Figure S5).^14^ Subtle quantitative differences for specific enzymes belonging to the above-mentioned functional classes were observed depending on *Trichoderma* BAM treatment; however, it was not possible to relate them to measured agronomic or nutritional characteristics.

On the other hand, an unexpected, reduced representation of proteins involved in RNA biosynthesis/processing (18 in number) or constituting large/small ribosomal subunits (26 in number) was detected in fruits from *Trichoderma* BAM-treated plants (Figures S6 and S7). This quantitative trend was particularly evident with the HA treatment and requires a further dedicated investigation in order to be understood. It may be tentatively considered in light of the concomitant reduced representation observed for a number of proteins involved in the stress response (65 in number), including those counteracting the detrimental action of ROS or facing the action of plant pathogens^70^ (Figure S4). Exceptions in this context were some heat shock proteins (HSP70 isoforms and HSP91) and some protein disulfide isomerase, DJ-1, and aldehyde dehydrogenase isoforms, which showed augmented levels after plant treatment. On the other hand, we observed the over-representation of proteins involved in vesicle trafficking, which are essential in the defense mechanisms of plant innate immunity and required to safely and timely deliver transport toxic antimicrobial compounds outside the cells (Figure S11).^71^ A modulation of proteins involved in defense response has already been demonstrated in root and leaf tissues of different plants treated with the living *Trichoderma* microbial preparations, which results in a coherent/incoherent quantitative trend of single proteins depending on the organism under investigation, the fungal formulation, and its timing of application.^27,39−42,44,45^

Our results also suggest that these fungal BAMs act as effector molecules in fungal–plant rhizosphere interactions, directly affecting the modulation of defense mechanisms systemically in peripheral plant organs (including fruits) and/or regulating concomitant ongoing ROS fluxes, thus confirming previous observations on tomato and *Lotus japonicus*.^27−29^ In the latter context, worth mentioning are the parallel reduced levels measured in this study for TAC, TPC, total and individual anthocyanins, as well as for antioxidant proteins (Table 2, Figures 1 and S3), which appear to generally indicate a ROS-enriched environment in the BAM-treated fruits. Slight quantitative differences for specific antioxidant proteins were observed depending on BAM treatment. Although the quantitative proteomic data on enzymes controlling the phenylpropanoid pathway at the beginning (phenylalanine-ammonia lyase and chorismate synthase) and subsequent (cinnamoyl-CoA reductase, cinnamate 4-hydrolase, chalcone and stilbene synthase, and cytochrome P450 98A2) processes supported the possible accumulation of naringenin chalcone and caffeic/ferulic acid derivatives in treated fruits, those on chalcone/flavone isomerase, elicitor-activated gene 3-1 and 3-2, and two isoflavone reductases indicated a metabolic block toward a continued production of the anthocyanin/lignin derivatives^72,73^ (Figure 4); the latter findings provided a rationale to the levels of total/individual anthocyanins measured in this study (Table 2, Figure 1). Augmented phenylalanine–ammonia lyase levels have already been reported in other tissues of other *Trichoderma*-treated plants.^38,41,74^ Finally, enzymes (geranylgeranyl reductase, hydroxymethylglutaryl-CoA synthase, diphosphomevalonate decarboxylase, squalene synthase, and β-amyrin synthase) involved in the biosynthesis of other secondary metabolites, namely, sequiterpenoids/triterpenoids, have also been found in increased levels after *Trichoderma* BAM applications (Figure 4). Many plant terpenoids, in particular the volatile compounds produced under biotic stress, may be useful in deterring pathogens or attracting their antagonists,^75−77^ thus inducing systemic resistance that primes defense responses also in other vegetative structures, such as fruits.^78,79^ Our data were in good agreement with those on corresponding genes and metabolites in tomato plants challenged with whole *T. harzianum* formulations^27^ and confirmed that treatment with BAMs can influence the plant defensive volatilome in strawberry. In the same context, the observed over-representation of other enzymes involved in the biosynthesis of other volatile aromatic compounds (pyruvate decarboxylase 2, acetyl-CoA carboxylase 1 and 2, and others) can be considered, including acids alcohols, esters, aldehydes, and fatty acids, which are responsible for the enhancement of strawberry flavor^37,80^ (Figures S3 and S5). Regarding specific proteins involved in plant response to pathogens that are able to elicit an allergenic effect on humans, this proteomic investigation unveiled preliminary information on the possible use of *Trichoderma* BAMs in reducing the content of seven known strawberry allergens in the fruits (Figure S4). However, this topic needs to be further investigated.

The above-reported agronomic and proteomic results suggested the activation of different signaling pathways and related down-stream processes in fruits after treatment with *Trichoderma* BAMs, similar to those observed in plants treated with formulations containing the entire living fungus.^27,38,81−85^ Indeed, signaling reactions based on Ca^2+^, jasmonic acid (JA), ethylene (ET), auxin, and brassinosteroid (BR) effectors were regulated in treated fruits, as proven in the observed changed levels of (i) topless-related proteins 1 and 3, WUS-interacting protein 2 and auxin transport protein (BIG) (showing over-representation); (ii) lipoxygenase 2, 12-oxophytodienoate reductase 2, peroxisomal 3-ketoacyl-CoA thiolase 3, and enoyl-CoA hydratase/isomerase family directly involved in JA biosynthesis (showing over-representation); (iii) a number of Ca^2+^-binding sensors and MAP3K (13 in number, mostly showing down-representation, except coherent Ca^2+^-translocating ATPase whose concentration ensured ion expulsion from ion-challenged cells); (iv) enzymes involved in Met conversion into ET (showing variable levels); and (v) BR-related membrane-associated progesterone-binding protein 3 (showing down-representation) (Figures 4 and S2). Subtle quantitative changes for some of the above-mentioned proteins were observed depending on *Trichoderma* BAM treatment. A number of these components participate in signaling reactions that ultimately determine the activation of cellular protein phosphorylation, ROS burst, and transcriptional reprogramming events, as well as the biosynthesis of various defense-related phytohormones. Although numerous protein level variations are similar to those observed in previous proteomic/transcriptomic investigations on other tissue samples collected from *Trichoderma*-treated plants,^27,38,40,44,45^ which suggest the activation of phytohormone-mediated signaling processes also in the case of BAM-treated strawberry, future investigations based on a simultaneous analysis of all diverse vegetative tissues from the *Trichoderma*-treated plants are required in order to clearly understand the time-course changes of Ca^2+^, JA, BR, and ET gradients following the microbial treatments. In fact, a number of effector concentration changes reported above are known to modulate plant growth and to proceed at different rates,^85,86^ and variable levels of these molecules have been reported in other tissues after fungal challenge.^28,87^

This study highlights the efficacy of the application of some *Trichoderma* BAMs to increase strawberry growth and yield, as well as some traits related to fruit quality. As expected, based on observed agronomical changes, the interaction between BAMs and strawberry induced changes in the fruit proteome. In particular, the applications of BAMs strongly modified the protein pattern related to fruit quality factors, carbon/energy metabolisms, and secondary metabolism. These results revealed changes in the abundance of specific proteins whose corresponding encoding genes have already been identified as deregulated in different plants receiving treatments with the whole organism in *Trichoderma* formulations as analyzed by dedicated transcriptomic studies.^27,45,74,76,77^ However, experimental limitations attributed to reduced protein level detection in gel-based proteomic hampered detailed proteomics in these previous investigations. Based on the present findings, it can be concluded that the use of selected *Trichoderma* metabolites can represent an innovative approach for improving strawberry cultivation.

## Supporting Information Available

HPLC-DAD chromatogram at 520 nm for compound identification; heat-map and hierarchical clustering of proteins involved in solute transport, calcium metabolism, and nutrient uptake; stress response; carbon and energy metabolism; amino acid metabolism, coenzyme metabolism, nucleotide metabolism, or lipid metabolism; RNA biosynthesis and RNA processing; protein biosynthesis; protein modification; protein translocation; protein degradation; vesicle trafficking, chromatin organization, DNA damage, and cell cycle; and cytoskeleton, cell wall, chromatin organization, DNA damage, and cell cycle; heat-map representation of hierarchical clustering analysis of proteins with unknown function; anthocyanins determined by LC-DAD-ESI-MS/MS analysis; identification and quantification details of the proteins; protein quantitative changes after BAM treatment; top-15 entries from functional enrichment analysis of strawberry DRPs; and bridged and nonlinked nodes identified during STRING analysis of DRPs (PDF)
Identification and quantification details of the proteins quantified in this study (XLSX)
Protein quantitative changes ascertained in fruits from plants treated with different Trichoderma BAMs, with respect to control (XLSX)

## Abbreviations

DRPs: differentially represented proteins
MRM: multiple reaction monitoring
MS: mass spectrometry
MS/MS: tandem mass spectrometry
ROS: reactive oxygen species
TMT: tandem mass tagging
