# Supplementary material for: Effect of Trichoderma Bioactive Metabolite Treatments on the Production, Quality, and Protein Profile of Strawberry Fruits
Source: J Agric Food Chem. 2020 May 19;68(27):7246–58. doi: 10.1021/acs.jafc.0c01438 (PMC8154561; doi:10.1021/acs.jafc.0c01438)

EFFECT OF *TRICHODERMA* BIOACTIVE METABOLITE TREATMENTS ON THE PRODUCTION, QUALITY AND PROTEIN PROFILE OF STRAWBERRY FRUITS.

Nadia Lombardi[1#]*, Anna Maria Salzano[2#], Antonio Dario Troise[1,2], Andrea Scaloni[2], Paola Vitaglione[1], Francesco Vinale[3,4], Roberta Marra[1], Simonetta Caira[2]*, Matteo Lorito[1,4,5], Giada d'Errico[1], Stefania Lanzuise[1] and Sheridan Lois Woo[4,5,6].

[1] Department of Agricultural Sciences, University of Naples Federico II, Portici, Naples, Italy

[2] Proteomics & Mass Spectrometry Laboratory, ISPAAM, National Research Council, Naples, Italy

[3] Department of Veterinary Medicine and Animal Productions, University of Naples Federico II, Naples, Italy

[4] Institute for Sustainable Plant Protection, National Research Council, Portici, Naples, Italy

[5] Task Force on Microbiome Studies, University of Naples Federico II, Naples, Italy

[6] Department of Pharmacy, University of Naples Federico II, Naples, Italy

# These authors contributed equally to this work

* Correspondence:
Dr. Nadia Lombardi
nadia.lombardi@unina.it

Dr. Simonetta Caira
simonetta.caira@cnr.it

SUPPLEMENTARY MATERIAL

Supplementary Figure S1. HPLC-DAD chromatogram of an exemplificative strawberry dried extract recorded at 520 nm. Putative compound identification was performed according to the literature data[6] and results reported in Supplementary Table S1. Typical pelargonidin and cyanidin moieties at m/z 271 and m/z 287 are reported in red and in black, respectively.

Supplementary Figure S2. Heat-map representation and hierarchical clustering analysis of proteins involved in solute transport (upper panel), calcium metabolism (middle panel) and nutrient uptake (lower panel), which were present in strawberry fruits produced by plants subjected to the treatment with different *Trichoderma* BAMs (HA, 6PP and HYTLO1), as compared to control (Ctrl). Shown are proteins presenting abundance fold changes ≥1.50 or ≤0.66 with respect to control (P≤ 0.05). Data are reported as $\log_2$ transformed abundance ratio values.

Supplementary Figure S3. Heat-map representation and hierarchical clustering analysis of proteins involved in stress response that were present in strawberry fruits produced by plants subjected to the treatment with different *Trichoderma* BAMs (HA, 6PP and HYTLO1), as compared to control (Ctrl). Shown are proteins presenting abundance fold changes ≥1.50 or ≤0.66 with respect to control (P≤ 0.05). Data are reported as $\log_2$ transformed abundance ratio values.

Supplementary Figure S4. Heat-map representation and hierarchical clustering analysis of proteins involved in carbon and energy metabolism that were present in strawberry fruits produced by plants subjected to the treatment with different *Trichoderma* BAMs (HA, 6PP and HYTLO1), as compared to control (Ctrl). Shown are proteins presenting abundance fold changes ≥1.50 or ≤0.66 with respect to control (P≤ 0.05). Data are reported as $\log_2$ transformed abundance ratio values.

Supplementary Figure S5. Heat-map representation and hierarchical clustering analysis of proteins involved in amino acid metabolism (upper panel), coenzyme metabolism (middle upper panel), nucleotide metabolism (middle panel) or lipid metabolism (middle lower panel), which were present in strawberry fruits produced by plants subjected to the treatment with different *Trichoderma* BAMs (HA, 6PP and HYTLO1), as compared to control (Ctrl). Shown are proteins presenting abundance fold changes ≥1.50 or ≤0.66 with respect to control (P≤ 0.05). Data are reported as $\log_2$ transformed abundance ratio values.

Supplementary Figure S6. Heat-map representation and hierarchical clustering analysis of proteins involved in RNA biosynthesis (upper panel) and RNA processing (lower panel), which

were present in strawberry fruits produced by plants subjected to the treatment with different *Trichoderma* BAMs (HA, 6PP and HYTLO1), as compared to control (Ctrl). Shown are proteins presenting abundance fold changes ≥1.50 or ≤0.66 with respect to control (P≤ 0.05). Data are reported as $\log_2$ transformed abundance ratio values.

Supplementary Figure S7. Heat-map representation and hierarchical clustering analysis of proteins involved in protein biosynthesis that were present in strawberry fruits produced by plants subjected to the treatment with different *Trichoderma* BAMs (HA, 6PP and HYTLO1), as compared to control (Ctrl). Shown are proteins presenting abundance fold changes ≥1.50 or ≤0.66 with respect to control (P≤ 0.05). Data are reported as $\log_2$ transformed abundance ratio values.

Supplementary Figure S8. Heat-map representation and hierarchical clustering analysis of proteins involved in protein modification, which were present in strawberry fruits produced by plants subjected to the treatment with different *Trichoderma* BAMs (HA, 6PP and HYTLO1), as compared to control (Ctrl). Shown are proteins presenting abundance fold changes ≥1.50 or ≤0.66 with respect to control (P≤ 0.05). Data are reported as $\log_2$ transformed abundance ratio values.

Supplementary Figure S9. Heat-map representation and hierarchical clustering analysis of proteins involved in protein translocation, which were present in strawberry fruits produced by plants subjected to the treatment with different *Trichoderma* BAMs (HA, 6PP and HYTLO1), as compared to control (Ctrl). Shown are proteins presenting abundance fold changes ≥1.50 or ≤0.66 with respect to control (P≤ 0.05). Data are reported as $\log_2$ transformed abundance ratio values.

Supplementary Figure S10. Heat-map representation and hierarchical clustering analysis of proteins involved in protein degradation, which were present in strawberry fruits produced by plants subjected to the treatment with different *Trichoderma* BAMs (HA, 6PP and HYTLO1), as compared to control (Ctrl). Shown are proteins presenting abundance fold changes ≥1.50 or ≤0.66 with respect to control (P≤ 0.05). Data are reported as $\log_2$ transformed abundance ratio values.

Supplementary Figure S11. Heat-map representation and hierarchical clustering analysis of proteins involved in vesicle trafficking that were present in strawberry fruits produced by plants subjected to the treatment with different *Trichoderma* BAMs (HA, 6PP and HYTLO1), as compared to control (Ctrl). Shown are proteins presenting abundance fold changes ≥1.50 or ≤0.66 with respect to control (P≤ 0.05). Data are reported as $\log_2$ transformed abundance ratio values.

Supplementary Figure S12. Heat-map representation and hierarchical clustering analysis of proteins involved in cytoskeleton (upper panel), cell wall (middle upper panel), chromatin organization (middle panel), DNA damage (middle lower panel) and cell cycle (lower panel), which were present in strawberry fruits produced by plants subjected to the treatment with different *Trichoderma* BAMs (HA, 6PP and HYTLO1), as compared to control (Ctrl). Shown are proteins presenting abundance fold changes ≥1.50 or ≤0.66 with respect to control (P≤ 0.05). Data are reported as log_2 transformed abundance ratio values.

Supplementary Figure S13. Heat-map representation of hierarchical clustering analysis of proteins with unknown function that were present in strawberry fruits produced by plants subjected to the treatment with different *Trichoderma* BAMs (HA, 6PP and HYTLO1), as compared to control (Ctrl). Shown are proteins presenting abundance fold changes ≥1.50 or ≤0.66 with respect to control (P≤ 0.05). Data are reported as log_2 transformed abundance ratio values.

Supplementary Table S1. Anthocyanins determined in strawberry samples by LC-DAD-ESI-MS/MS analysis, including peak number, retention time (RT), precursor and product ions, their tentative identification and abbreviation.

Peak	RT (min)	Precursor ion $[M]^+$	Product ion	Tentative identification	Abbreviation
1	11.2	449	287	cyanidin 3-*O*-glucoside	cya 3-*O*-glc
2	12.8	433	271	pelargonidin 3-*O*-glucoside	pel 3-*O*-glc
3	13.5	579	271	pelargonidin 3-*O*-rutinoside	pel 3-*O*-rut
4	14.5	519	271	pelargonidin 3-*O*-malonyl-glucoside	pel 3-*O*-mal-glc
5	15.1	475	271	pelargonidin 3-*O*-acetyl-glucoside	pel 3-*O*-ac-glc
6	17.0	449	287	cyanidin derivative	cya der

Supplementary Table S2. Identification and quantification details of the proteins examined in this study. Protein accession and description, exp. *q*-value, sum posterior error probability (PEP) score, sequence coverage (%), number of identified peptides, peptide spectrum matches (PSMs), protein groups, number of identified unique peptides, number of razor peptides and Mascot identification score are reported, together with protein theoretical values (number of amino acids, molecular mass, pI). Specific information on protein quantification are reported including: Found in file, Found in sample, modification(s), abundance ratios, abundance (grouped), abundance (scaled), abundance, abundance (normalized). TAIR accession number for homologues counterparts in *Arabidopsis thaliana* and results of BLAST alignments are reported for the identified proteins.

Supplementary Table S3. Protein quantitative changes ascertained in fruits from plants treated with different *Trichoderma* BAMs (HA, 6PP and HYTLO1), with respect to control. Functional assignment of proteins was performed as reported in the experimental section. Figures containing resulting outputs are also reported.

Supplementary Table S4. Top-15 entries deriving from functional enrichment analysis of strawberry DRPs after treatment with different *Trichoderma* BAMs (HA, 6PP and HYTLO1). Results from Biological process (GO), Molecular function (GO) and KEGG pathways enrichment are shown.

**Biological Process (GO)**			
GO-term	*Description*	*Count in gene set*	*False discovery rate*
GO:0010038	response to metal ion	63 of 414	3.87e-31
GO:0010035	response to inorganic substance	83 of 795	3.87e-31
GO:0046686	response to cadmium ion	54 of 286	6.15e-31
GO:0043603	cellular amide metabolic process	65 of 625	3.75e-24
GO:0006518	peptide metabolic process	55 of 513	8.62e-21
GO:0050896	response to stimulus	195 of 5064	1.27e-20
GO:0043604	amide biosynthetic process	54 of 501	1.27e-20
GO:1901566	organonitrogen compound biosynthetic process	82 of 1229	2.03e-19
GO:0055114	oxidation-reduction process	85 of 1348	8.86e-19
GO:0006412	translation	48 of 433	8.86e-19
GO:0044281	small molecule metabolic process	90 of 1503	1.08e-18
GO:0042221	response to chemical	125 of 2654	1.84e-18
GO:0009987	cellular process	310 of 10581	1.84e-18
GO:0006950	response to stress	131 of 2932	1.17e-17
GO:0009628	response to abiotic stimulus	93 of 1699	4.48e-17

**Molecular Function (GO)**			
GO-term	*Description*	*Count in gene set*	*False discovery rate*
GO:0005488	binding	276 of 8611	3.26e-20
GO:0003824	catalytic activity	238 of 7239	1.42e-17
GO:0016491	oxidoreductase activity	76 of 1201	1.24e-16
GO:0043167	ion binding	183 of 5070	3.17e-16
GO:0043169	cation binding	120 of 2949	5.78e-13
GO:0046872	metal ion binding	119 of 2940	9.38e-13
GO:0005198	structural molecule activity	37 of 415	8.61e-12

GO:0036094	small molecule binding	107 of 2633	1.81e-11
GO:0043168	anion binding	106 of 2629	3.47e-11
GO:0016874	ligase activity	24 of 185	1.24e-10
GO:0003735	structural constituent of ribosome	29 of 309	8.82e-10
GO:0000166	nucleotide binding	95 of 2461	5.58e-09
GO:0005507	copper ion binding	20 of 157	8.39e-09
GO:0046914	transition metal ion binding	50 of 933	1.12e-08
GO:0032553	ribonucleotide binding	87 of 2204	1.12e-08

KEGG Pathways

Pathway	*Description*	*Count in gene set*	*False discovery rate*
ath01100	Metabolic pathways	115 of 1899	2.33e-25
ath01110	Biosynthesis of secondary metabolites	72 of 1063	7.75e-18
ath04141	Protein processing in endoplasmic reticulum	29 of 205	3.74e-14
ath00190	Oxidative phosphorylation	22 of 149	3.74e-11
ath03010	Ribosome	28 of 318	2.45e-09
ath01200	Carbon metabolism	25 of 261	4.02e-09
ath00970	Aminoacyl-tRNA biosynthesis	12 of 57	7.90e-08
ath00010	Glycolysis / Gluconeogenesis	15 of 115	3.00e-07
ath00620	Pyruvate metabolism	13 of 85	4.25e-07
ath04144	Endocytosis	14 of 142	1.56e-05
ath03050	Proteasome	9 of 58	3.86e-05
ath00480	Glutathione metabolism	11 of 98	5.45e-05
ath00710	Carbon fixation in photosynthetic organisms	9 of 69	0.00012
ath03040	Spliceosome	14 of 184	0.00017
ath00592	alpha-Linolenic acid metabolism	7 of 41	0.00019

Supplementary Table S5. Bridged and non-linked nodes identified during STRING analysis of DRPs present in strawberry fruits after plant treatment with different *Trichoderma* BAMs (HA, 6PP and HYTLO1). Functional protein association was based on data recorded for *A. thaliana* protein homologues, whose name is provided.

**TAIR entry**	**Symbol**	**Description**
AT1G01470	LEA14	Probable desiccation-related protein LEA14;
AT1G01800	AT1G01800	NAD(P)-binding Rossmann-fold superfamily protein;
AT1G02080	AT1G02080	Transcription regulator;
AT1G03860	PHB2	Prohibitin-2, mitochondrial;
AT1G04170	F20D22.6	Similar to initiation factor eIF-2;
AT1G04430	AT1G04430	S-adenosyl-L-methionine-dependent methyltransferases superfamily protein;
AT1G04510	MAC3A	Pre-mRNA-processing factor 19 homolog 1;
AT1G04760	VAMP726	Putative vesicle-associated membrane protein 726;
AT1G04980	PDIL2-2	Protein disulfide isomerase-like (PDIL) protein 2-2;
AT1G05010	EFE	1-Aminocyclopropane-1-carboxylate oxidase 4;
AT1G06220	MEE5	Ribosomal protein S5/Elongation factor G/III/V family protein;
AT1G06410	TPS7	Probable alpha,alpha-trehalose-phosphate synthase [UDP-forming] 7;
AT1G06950	TIC110	Translocon at the inner envelope membrane of chloroplasts 110;
AT1G07810	ECA1	Calcium-transporting ATPase 1, endoplasmic reticulum-type;
AT1G07890	APX1	Cytosolic ascorbate peroxidase APX1;
AT1G08830	CSD1	Cytosolic copper/zinc superoxide dismutase CSD1;
AT1G09020	SNF4	Sucrose non-fermenting protein 4;
AT1G09080	BIP3	Probable mediator of RNA polymerase II transcription subunit 37b;
AT1G09210	CRT1b	Calreticulin 1b;
AT1G09620	AT1G09620	Leucine-tRNA ligase, cytoplasmic;
AT1G09760	U2A	U2 small nuclear ribonucleoprotein A;
AT1G10950	TMN1	Transmembrane 9 superfamily member 1;
AT1G11760	MED32	Mediator of RNA polymerase II transcription subunit 32;
AT1G12310	AT1G12310	Calcium-binding EF-hand family protein;

AT1G12410	CLP2	ATP-dependent Clp protease subunit-related protein 2, chloroplastic;
AT1G12640	LPLAT1	MBOAT (membrane bound O-acyl transferase) family protein;
AT1G13440	GAPC2	Glyceraldehyde-3-phosphate dehydrogenase GAPC2, cytosolic;
AT1G13700	PGL1	Probable 6-phosphogluconolactonase 1;
AT1G14610	TWN2	Valyl-tRNA synthetase/valine-tRNA ligase (VALRS);
AT1G15520	ABCG40	ABC transporter G family member 40;
AT1G15690	AVP1	Pyrophosphate-energized vacuolar membrane proton pump 1;
AT1G16030	Hsp70b	Heat shock 70 kDa protein 5;
AT1G16780	VHP2;2	Pyrophosphate-energized membrane proton pump 3;
AT1G17260	AHA10	Autoinhibited H(+)-ATPase isoform 10;
AT1G17730	VPS46.1	Vacuolar protein sorting 46.1;
AT1G17880	BTF3	Basic transcription factor 3 (BTF3);
AT1G18270	AT1G18270	Ketose-bisphosphate aldolase class-II family protein;
AT1G20200	EMB2719	26S proteasome non-ATPase regulatory subunit 3 homolog A;
AT1G20330	SMT2	24-Methylenesterol C-methyltransferase 2;
AT1G20950	AT1G20950	Pyrophosphate-fructose 6-phosphate 1-phosphotransferase subunit alpha 1;
AT1G21750	PDIL1-1	Protein disulfide isomerase-like (PDIL) protein 1-1;
AT1G23100	AT1G23100	Putative 10kDa chaperonin (CPN10) protein;
AT1G23740	AOR	NADPH-dependent alkenal/one oxidoreductase, chloroplastic;
AT1G24100	UGT74B1	UDP-glucosyl transferase 74B1;
AT1G25350	OVA9	Glutamine-tRNA ligase, putative / glutaminyl-tRNA synthetase, putative/GlnRS;
AT1G25480	AT1G25480	Aluminium activated malate transporter family protein;
AT1G26110	DCP5	Protein decapping 5;
AT1G26340	CB5-A	Cytochromes b5 family-protein present in the chloroplast envelope;
AT1G26880	AT1G26880	Ribosomal protein L34e superfamily protein;
AT1G27310	NTF2A	Nuclear transport factor 2A;
AT1G29880	AT1G29880	Glycyl-tRNA synthetase/glycine-tRNA ligase;
AT1G29900	CARB	Carbamoyl-phosphate synthase large chain, chloroplastic;
AT1G29990	PFD6	Prefoldin 6;
AT1G30070	AT1G30070	SGS domain-containing protein;
AT1G31480	SGR2	Shoot gravitropism 2 (SGR2);
AT1G31812	ACBP6	Acyl-CoA-binding domain-containing protein 6;

AT1G31850	AT1G31850	S-adenosyl-L-methionine-dependent methyltransferases superfamily protein;
AT1G32790	CID11	Polyadenylate-binding protein-interacting protein 11;
AT1G33140	PGY2	Ribosomal protein L9;
AT1G35620	PDIL5-2	Protein disulfide-isomerase 5-2;
AT1G36160	ACC1	Acetyl-CoA carboxylase 1;
AT1G36180	ACC2	Acetyl-CoA carboxylase 2;
AT1G41830	SKS6	SKU5-similar 6 (SKS6);
AT1G43700	VIP1	VIRE2-interacting protein 1;
AT1G47550	SEC3A	Exocyst complex component SEC3A;
AT1G47710	SERPIN1	Serine protease inhibitor (SERPIN) family protein;
AT1G48410	AGO1	Stabilizer of iron transporter SufD/Polynucleotidyl transferase;
AT1G48830	AT1G48830	Ribosomal protein S7e family protein;
AT1G48850	EMB1144	Chorismate synthase, 5-enolpyruvylshikimate-3-phosphate phospholyase;
AT1G49340	PI4KA1	Phosphatidylinositol 3- and 4-kinase family protein;
AT1G50200	ALATS	Alanyl-tRNA synthetase;
AT1G50380	AT1G50380	Prolyl oligopeptidase family protein;
AT1G50500	HIT1	Vacuolar protein sorting-associated protein 53 A;
AT1G51590	MNS1	Mannosyl-oligosaccharide 1,2-alpha-mannosidase MNS1;
AT1G51710	UBP6	Ubiquitin carboxyl-terminal hydrolase 6;
AT1G51980	AT1G51980	Probable mitochondrial-processing peptidase subunit alpha-1, mitochondrial;
AT1G52570	PLDALPHA2	Phospholipase D alpha 2;
AT1G53280	DJ1B	Class I glutamine amidotransferase-like superfamily protein;
AT1G53310	PPC1	Phosphoenolpyruvate carboxylase 1;
AT1G53540	AT1G53540	HSP20-like chaperone-superfamily protein;
AT1G54100	ALDH7B4	Aldehyde dehydrogenase family 7 member B4;
AT1G54170	CID3	Polyadenylate-binding protein-interacting protein 3;
AT1G54270	EIF4A-2	Eukaryotic initiation factor 4A-2;
AT1G54870	AT1G54870	NADPH-dependent aldehyde reductase 1, chloroplastic;
AT1G55260	AT1G55260	Bifunctional inhibitor/lipid-transfer protein/seed storage 2S albumin;
AT1G56070	LOS1	Ribosomal protein S5/Elongation factor G/III/V family protein;
AT1G59960	AT1G59960	NAD(P)-linked oxidoreductase superfamily protein;
AT1G60070	AT1G60070	Adaptor protein complex AP-1, gamma subunit;

AT1G60420	AT1G60420	DC1 domain-containing protein;
AT1G60500	DRP4C	Dynamin related protein 4C;
AT1G60690	AT1G60690	NAD(P)-linked oxidoreductase superfamily protein;
AT1G60710	ATB2	NAD(P)-linked oxidoreductase superfamily protein;
AT1G61150	AT1G61150	LisH and RanBPM domains containing protein;
AT1G62020	AT1G62020	Coatomer subunit alpha-1;
AT1G63220	AT1G63220	Calcium-dependent lipid-binding (CaLB domain) family protein;
AT1G63800	UBC5	Ubiquitin-conjugating enzyme E2 5;
AT1G64040	TOPP3	Serine/threonine-protein phosphatase PP1 isozyme 3;
AT1G64390	GH9C2	Glycosyl hydrolase 9C2 (GH9C2);
AT1G64660	MGL	Methionine gamma-lyase;
AT1G64790	ILA	ILITYHIA (ILA) HEAT repeat protein involved in plant immunity;
AT1G65260	PTAC4	Membrane-associated protein VIPP1, chloroplastic;
AT1G65930	cICDH	Cytosolic NADP+-dependent isocitrate dehydrogenase;
AT1G65980	TPX1	Thioredoxin-dependent peroxidase 1;
AT1G66240	At1g66240	Copper transport protein ATX1;
AT1G66680	AR401	S-adenosyl-L-methionine-dependent methyltransferase superfamily protein;
AT1G66950	ABCG39	ABC transporter G family member 39;
AT1G70600	AT1G70600	Ribosomal protein L18e/L15 superfamily protein;
AT1G71220	EBS1	UDP-glucose:glycoprotein glucosyltransferase;
AT1G71820	SEC6	Exocyst complex component SEC6;
AT1G71950	AT1G71950	Uncharacterized protein At1g71950;
AT1G72160	AT1G72160	Sec14p-like phosphatidylinositol transfer family protein;
AT1G72330	ALAAT2	Alanine aminotransferase ALAAT2;
AT1G72960	AT1G72960	Root hair defective 3 GTP-binding protein (RHD3);
AT1G73370	SUS6	Sucrose synthase 6;
AT1G74050	AT1G74050	Ribosomal protein L6 family protein;
AT1G74380	XXT5	Probable xyloglucan 6-xylosyltransferase 5;
AT1G74910	AT1G74910	ADP-glucose pyrophosphorylase family protein;
AT1G74920	ALDH10A8	Similar to betaine aldehyde dehydrogenase;
AT1G75220	ERDL6	Major facilitator superfamily protein;
AT1G75270	DHAR2	Glutathione S-transferase DHAR2;

AT1G75280	AT1G75280	NmrA-like negative transcriptional regulator family protein;
AT1G75950	SKP1	S phase kinase-associated protein 1;
AT1G76160	sks5	AT1G76160 protein; SKU5 similar 5 (sks5);
AT1G76550	AT1G76550	Pyrophosphate--fructose 6-phosphate 1-phosphotransferase subunit alpha 2;
AT1G76690	OPR2	12-Oxophytodienoate reductase 2;
AT1G77120	ADH1	Alcohol dehydrogenase class-P;
AT1G77490	TAPX	Chloroplastic thylakoid ascorbate peroxidase tAPX;
AT1G77550	AT1G77550	Tubulin-tyrosine ligase;
AT1G77700	AT1G77700	Pathogenesis-related thaumatin superfamily protein;
AT1G78380	GSTU19	Glutathione transferase of Tau GST gene family;
AT1G78570	RHM1	Rhamnose biosynthesis protein 1;
AT1G78870	UBC35	Ubiquitin-conjugating enzyme E2 35;
AT1G78950	BAS	Terpenoid cyclases family protein;
AT1G79650	RAD23B	Rad23 UV excision repair protein family;
AT1G79690	NUDT3	Nudix hydrolase homolog 3;
AT1G79930	HSP91	Heat shock 70 kDa protein 14;
AT1G80070	EMB14	Pre-mRNA-processing-splicing factor 8A;
AT1G80230	AT1G80230	Cytochrome c oxidase subunit 5b-2, mitochondrial;
AT1G80300	NTT1	ADP,ATP carrier protein 1, chloroplastic;
AT1G80360	AT1G80360	Pyridoxal phosphate-dependent transferases superfamily protein;
AT1G80490	T21F11.18	Topless-related protein 1;
AT2G01530	MLP329	MLP-like protein 329 (MLP329);
AT2G01690	AT2G01690	ARM repeat superfamily protein;
AT2G01720	AT2G01720	Dolichyl-diphosphooligosaccharide--protein glycosyltransferase subunit 1B;
AT2G02010	GAD4	Glutamate decarboxylase 4;
AT2G02560	CAND1	CAND1 (cullin-associated and neddylation-dissociated) protein;
AT2G05170	VPS11	Homologous to yeast VPS11.
AT2G05710	ACO3	Aconitate hydratase 3, mitochondrial;
AT2G05990	MOD1	Enoyl-[acyl-carrier-protein] reductase [NADH], chloroplastic;
AT2G13560	NAD-ME1	NAD-dependent malic enzyme (NAD-ME) not acting on oxaloacetate;
AT2G14740	VSR3	Vaculolar sorting receptor 3;
AT2G15220	AT2G15220	Plant basic secretory protein (BSP) family protein;

AT2G16060	HB1	Non-symbiotic hemoglobin 1;
AT2G16600	ROC3	Peptidyl-prolyl cis-trans isomerase CYP19-1;
AT2G17390	AKR2B	Ankyrin repeat domain-containing protein 2B;
AT2G17980	ATSLY1	Sec1/munc18-like (SM) proteins superfamily;
AT2G18110	AT2G18110	Translation elongation factor EF1B/ribosomal protein S6;
AT2G19520	FVE	Transducin family protein/WD-40 repeat family protein;
AT2G20360	AT2G20360	NADH dehydrogenase [ubiquinone] 1 alpha subcomplex subunit 9, mitochondrial;
AT2G20450	AT2G20450	Ribosomal protein L14;
AT2G20560	AT2G20560	DNAJ heat shock family protein;
AT2G20580	RPN1A	26S proteasome non-ATPase regulatory subunit 2 homolog A;
AT2G20760	AT2G20760	Clathrin light chain protein;
AT2G21790	RNR1	Ribonucleoside-diphosphate reductase large subunit;
AT2G21870	MGP1	Copper/cobalt/zinc mitochondrial membrane ATP synthase (F(1)F(0);
AT2G22400	AT2G22400	S-adenosyl-L-methionine-dependent methyltransferases superfamily protein;
AT2G23420	NAPRT2	Nicotinate phosphoribosyltransferase 2;
AT2G24270	ALDH11A3	Non-phosphorylating NADP-dependent glyceraldehyde-3-phosphate dehydrogenase
AT2G24520	HA5	ATPase 5, plasma membrane-type;
AT2G25670	AT2G25670	Uncharacterized protein At2g25670;
AT2G25970	AT2G25970	Uncharacterized protein At2g25970;
AT2G26080	GLDP2	Glycine dehydrogenase (decarboxylating) 2, mitochondrial;
AT2G26590	RPN13	26S proteasome regulatory subunit RPN13;
AT2G26890	GRV2	DNAJ heat shock N-terminal domain-containing protein;
AT2G28000	CPN60A	Chaperonin 60 subunit alpha 1, chloroplastic;
AT2G29500	AT2G29500	HSP20-like chaperones superfamily protein;
AT2G30110	UBA1	Ubiquitin-activating enzyme (E1);
AT2G30490	C4H	Trans-cinnamate 4-monooxygenase;
AT2G30860	GSTF9	Glutathione S-transferase PHI 9;
AT2G31660	SAD2	ARM repeat superfamily protein;
AT2G32520	AT2G32520	Alpha/beta-hydrolase superfamily protein;
AT2G32730	AT2G32730	26S proteasome regulatory complex, non-ATPase subcomplex, Rpn2/Psmd1 subunit;
AT2G33150	PKT3	3-Ketoacyl-CoA thiolase 2, peroxisomal;
AT2G33410	AT2G33410	RNA-binding (RRM/RBD/RNP motifs) family protein;

AT2G33870	ArRABA1h	Ras-related protein RABA1h;
AT2G34160	AT2G34160	Uncharacterized protein At2g34160;
AT2G35050	AT2G35050	Protein kinase superfamily protein with octicosapeptide/Phox/Bem1p domain;
AT2G35120	AT2G35120	Glycine cleavage system H protein 2, mitochondrial;
AT2G36060	MMZ3	Ubiquitin-conjugating enzyme E2 variant 1C;
AT2G36070	TIM44-2	Mitochondrial import inner membrane translocase subunit TIM44-2;
AT2G36380	ABCG34	ABC transporter G family member 34;
AT2G36620	RPL24A	RPL24A - ribosomal protein L24;
AT2G37170	PIP2B	Plasma membrane intrinsic protein 2;
AT2G37190	AT2G37190	Ribosomal protein L11 family protein;
AT2G37270	RPS5B	Ribosomal protein S5 isoform;
AT2G38280	FAC1	AMP deaminase, putative/myoadenylate deaminase, putative;
AT2G39390	AT2G39390	Ribosomal L29 family protein;
AT2G40010	AT2G40010	Ribosomal protein L10 family protein;
AT2G40300	FER4	Ferritin-4, chloroplastic;
AT2G40800	AT2G40800	Import inner membrane translocase subunit;
AT2G40890	CYP98A3	Cytochrome P450, family 98, subfamily A, polypeptide 3;
AT2G41620	AT2G41620	Nucleoporin interacting component (Nup93/Nic96-like) family protein;
AT2G41680	NTRC	NADPH-dependent thioredoxin reductase 3;
AT2G41740	VLN2	Villin-2;
AT2G41790	AT2G41790	Insulinase (Peptidase family M16) family protein;
AT2G42680	MBF1A	Multiprotein-bridging factor 1a;
AT2G43970	LARP6b	La-related protein 6B;
AT2G44060	AT2G44060	Late embryogenesis abundant protein, group 2;
AT2G44310	AT2G44310	Calcium-binding EF-hand family protein;
AT2G44350	ATCS	Mitochrondrion targeted citrate synthase;
AT2G46520	AT2G46520	Cellular apoptosis susceptibility protein, putative/importin-alpha re-exporter;
AT2G47470	UNE5	Protein disulfide isomerase-like (PDIL) protein;
AT2G47730	GSTF8	Glutathione S-transferase F8, chloroplastic;
AT3G01280	VDAC1	Mitochondrial outer membrane protein porin 1;
AT3G01390	VMA10	V-type proton ATPase subunit G1;
AT3G01640	GLCAK	Glucuronokinase 1;

AT3G01680	SEOR1	Protein sieve element occlusion B;
AT3G01780	TPLATE	ARM repeat superfamily protein;
AT3G02090	MPPBETA	Probable mitochondrial-processing peptidase subunit beta, mitochondrial;
AT3G02260	BIG	Auxin transport protein (BIG);
AT3G02540	RAD23C	Rad23 UV excision repair protein family;
AT3G02540	RAD23C	Rad23 UV excision repair protein family;
AT3G02720	DJ1D	Class I glutamine amidotransferase-like superfamily protein;
AT3G02760	AT3G02760	Class II aaRS and biotin synthetases superfamily protein;
AT3G03380	DEG7	Protease Do-like 7;
AT3G03480	CHAT	Acetyl CoA:(Z)-3-hexen-1-ol acetyltransferase;
AT3G04120	GAPC1	Glyceraldehyde-3-phosphate dehydrogenase GAPC1, cytosolic;
AT3G04650	AT3G04650	FAD/NAD(P)-binding oxidoreductase family protein;
AT3G05040	HASTY	ARM repeat superfamily protein;
AT3G05560	AT3G05560	Ribosomal L22e protein family;
AT3G06050	PRXIIF	Peroxiredoxin-2F, mitochondrial;
AT3G06860	MFP2	Peroxisomal fatty acid beta-oxidation multifunctional protein MFP2;
AT3G07020	SGT	UDP-Glycosyltransferase superfamily protein;
AT3G07100	ERMO2	Protein transport protein Sec24-like At3g07100;
AT3G07660	AT3G07660	Kinase-related protein of unknown function (DUF1296);
AT3G07810	AT3G07810	RNA-binding (RRM/RBD/RNP motifs) family protein;
AT3G07810	AT3G07810	RNA-binding (RRM/RBD/RNP motifs) family protein;
AT3G08510	PLC2	Phosphoinositide phospholipase C 2;
AT3G09270	GSTU8	Glutathione S-transferase TAU 8;
AT3G09980	AT3G09980	Ankyrin repeat 30A-like protein (DUF662);
AT3G10640	VPS60.1	Vacuolar protein sorting-associated protein 60.1;
AT3G10920	MSD1	Superoxide dismutase [Mn] 1, mitochondrial;
AT3G11050	FER2	Ferritin-2, chloroplastic;
AT3G11130	AT3G11130	Clathrin heavy chain 1;
AT3G11400	EIF3G1	Eukaryotic translation initiation factor 3 subunit G;
AT3G11830	AT3G11830	TCP-1/cpn60 chaperonin family protein;
AT3G11910	UBP13	Ubiquitin carboxyl-terminal hydrolase 13;
AT3G12390	AT3G12390	Nascent polypeptide-associated complex (NAC), alpha subunit family protein;

AT3G12490	CYSB	Cysteine proteinase inhibitor 6;
AT3G12580	HSP70	Probable mediator of RNA polymerase II transcription subunit 37c;
AT3G12915	AT3G12915	Ribosomal protein S5/Elongation factor G/III/V family protein;
AT3G13224	AT3G13224	RNA-binding (RRM/RBD/RNP motifs) family protein;
AT3G13330	PA200	Proteasome activating protein 200;
AT3G14940	PPC3	Cytosolic phosphoenolpyruvate carboxylase;
AT3G15660	GRX4	Monothiol glutaredoxin-S15, mitochondrial;
AT3G15730	PLDALPHA1	Phospholipase D alpha 1;
AT3G15880	WSIP2	Topless-related protein 4;
AT3G16640	TCTP	Translationally controlled tumor protein;
AT3G16760	AT3G16760	Tetratricopeptide repeat (TPR)-like superfamily protein;
AT3G16810	PUM24	Pumilio homolog 24;
AT3G17390	MTO3	S-adenosylmethionine synthetase family protein;
AT3G17880	TDX	Tetraticopeptide domain-containing thioredoxin;
AT3G18060	AT3G18060	Transducin family protein/WD-40 repeat family protein;
AT3G18860	AT3G18860	Transducin family protein/WD-40 repeat family protein;
AT3G19000	AT3G19000	2-oxoglutarate (2OG) and Fe(II)-dependent oxygenase superfamily protein;
AT3G19240	AT3G19240	Vacuolar import/degradation, Vid27-related protein;
AT3G20390	AT3G20390	Reactive Intermediate Deaminase A, chloroplastic;
AT3G22630	PBD1	Proteasome subunit beta type-2-A;
AT3G24350	SYP32	Syntaxin of plants 32;
AT3G25150	AT3G25150	Nuclear transport factor 2 with RNA binding (RRM-RBD-RNP motifs) domain;
AT3G25520	RPL5A	60S ribosomal protein L5-1;
AT3G25580	AT3G25580	Thioredoxin superfamily protein;
AT3G25800	PP2AA2	Serine/threonine-protein phosphatase 2A 65 kDa regulatory subunit A beta;
AT3G27530	GC6	Golgin candidate 6;
AT3G27740	CARA	Carbamoyl-phosphate synthase small chain, chloroplastic;
AT3G42050	AT3G42050	Vacuolar ATP synthase subunit H family protein;
AT3G42170	DAYSLEEPER	Zinc finger BED domain-containing protein DAYSLEEPER;
AT3G42640	HA8	ATPase 8, plasma membrane-type;
AT3G43300	ATMIN7	Brefeldin A-inhibited guanine nucleotide-exchange protein 5;
AT3G43810	CAM7	Calmodulin-7;

AT3G44590	AT3G44590	60S acidic ribosomal protein family;
AT3G45140	LOX2	Lipoxygenase 2, chloroplastic;
AT3G45600	TET3	Tetraspanin-3;
AT3G46740	TOC75-III	Component of the translocon outer membrane (TOC) complex;
AT3G46970	PHS2	Alpha-glucan phosphorylase 2, cytosolic;
AT3G47950	HA4	ATPase 4, plasma membrane-type;
AT3G48000	ALDH2B4	Aldehyde dehydrogenase family 2 member B4, mitochondrial;
AT3G48170	ALDH10A9	Putative betaine aldehyde dehydrogenase;
AT3G48680	GAMMACAL2	Gamma carbonic anhydrase-like protein;
AT3G48890	MAPR3	Putative progesterone-binding protein homolog (Atmp2) mRNA;
AT3G49010	BBC1	60S ribosomal protein L13;
AT3G49470	NACA2	Nascent polypeptide-associated complex subunit alpha-like protein 2;
AT3G49910	AT3G49910	Translation protein SH3-like family protein;
AT3G50590	AT3G50590	Transducin/WD40 repeat-like superfamily protein;
AT3G51000	AT3G51000	Alpha/beta-hydrolase superfamily protein;
AT3G51730	AT3G51730	Saposin B domain-containing protein;
AT3G51810	EM1	Stress induced protein;
AT3G52140	NOXY38	Tetratricopeptide repeat (TPR)-containing protein;
AT3G52300	ATPQ	ATP synthase subunit d, mitochondrial;
AT3G52730	AT3G52730	Ubiquinol-cytochrome C reductase UQCRX/QCR9-like family protein;
AT3G52950	AT3G52950	CBS/octicosapeptide/Phox/Bemp1 (PB1) domains-containing protein;
AT3G52990	AT3G52990	Pyruvate kinase family protein;
AT3G53020	STV1	RPL24B encodes ribosomal protein L24,
AT3G53260	PAL2	Phenylalanine ammonia-lyase 2;
AT3G53420	PIP2A	Plasma membrane intrinsic protein PIP2;
AT3G53990	AT3G53990	Adenine nucleotide alpha hydrolases-like superfamily protein;
AT3G54250	AT3G54250	Diphosphomevalonate decarboxylase MVD2, peroxisomal;
AT3G54440	AT3G54440	Glycoside hydrolase family 2 protein;
AT3G54820	PIP2;5	Plasma membrane intrinsic protein 2;5;
AT3G55120	TT5	Chalcone-flavanone isomerase family protein;
AT3G55200	SAP130a	Cleavage and polyadenylation specificity factor (CPSF) A subunit;
AT3G55410	AT3G55410	2-Oxoglutarate dehydrogenase, E1 component;

AT3G55610	P5CS2	Delta-1-pyrroline-5-carboxylate synthase B;
AT3G56070	ROC2	Peptidyl-prolyl cis-trans isomerase CYP19-3;
AT3G56460	AT3G56460	GroES-like zinc-binding alcohol dehydrogenase family protein;
AT3G57520	SIP2	Probable galactinol--sucrose galactosyltransferase 2;
AT3G58680	MBF1B	Multiprotein-bridging factor 1b;
AT3G60860	AT3G60860	Brefeldin A-inhibited guanine nucleotide-exchange protein 2;
AT3G61860	RS31	RNA-binding (RRM/RBD/RNP motifs) family protein;
AT3G62120	AT3G62120	Class II aaRS and biotin synthetase superfamily protein;
AT3G62840	F17A22.3	Small nuclear ribonucleoprotein family protein;
AT3G62870	AT3G62870	Ribosomal protein L7Ae/L30e/S12e/Gadd45 family protein;
AT3G63460	AT3G63460	Transducin family protein/WD-40 repeat family protein;
AT4G00100	RPS13A	Cytoplasmic ribosomal protein S13 involved in leaf development;
AT4G00430	PIP1;4	Plasma membrane intrinsic protein 1;4;
AT4G01320	ATSTE24	Peptidase family M48 family protein;
AT4G01850	SAM-2	S-adenosylmethionine synthetase 2;
AT4G01900	GLB1	Nitrogen regulatory protein P-II homolog;
AT4G02080	SAR2	Secretion-associated RAS super family 2;
AT4G02350	SEC15B	Exocyst complex component SEC15B;
AT4G02570	CUL1	Cullin-1;
AT4G02620	AT4G02620	Vacuolar ATPase subunit F family protein;
AT4G03240	FH	Frataxin, mitochondrial;
AT4G04020	FIB	Probable plastid-lipid-associated protein 1, chloroplastic;
AT4G05020	NDB2	External alternative NAD(P)H-ubiquinone oxidoreductase B2, mitochondrial;
AT4G05050	UBQ11	Polyubiquitin 11;
AT4G05160	AT4G05160	AMP-dependent synthetase and ligase family protein;
AT4G05420	DDB1A	Damaged DNA binding protein 1A;
AT4G09020	ISA3	Isoamylase 3, chloroplastic;
AT4G09320	NDPK1	Nucleoside diphosphate kinase family protein;
AT4G09800	RPS18C	40S ribosomal protein S18;
AT4G10020	HSD5	11-Beta-hydroxysteroid dehydrogenase-like 5;
AT4G10320	AT4G10320	tRNA synthetase class I (I, L, M and V) family protein;
AT4G11420	EIF3A	Eukaryotic translation initiation factor 3 subunit A;

AT4G11600	GPX6	Probable phospholipid hydroperoxide glutathione peroxidase 6, mitochondrial;
AT4G11740	SAY1	Plant UBX domain-containing protein 8;
AT4G11820	HMGS	Hydroxymethylglutaryl-CoA synthase;
AT4G12590	AT4G12590	Protein of unknown function DUF106, transmembrane;
AT4G12770	AT4G12770	Chaperone DnaJ-domain superfamily protein;
AT4G13200	AT4G13200	Uncharacterized protein At4g13200, chloroplastic;
AT4G13780	AT4G13780	Methionine-tRNA ligase;
AT4G13940	HOG1	S-adenosyl-L-homocysteine hydrolase;
AT4G14710	ATARD2	1,2-Dihydroxy-3-keto-5-methylthiopentene dioxygenase 3;
AT4G15480	UGT84A1	UDP-glycosyltransferase superfamily protein;
AT4G16130	ARA1	Arabinose kinase;
AT4G16720	AT4G16720	Ribosomal protein L23/L15e family protein;
AT4G17140	AT4G17140	Pleckstrin homology (PH) domain-containing protein;
AT4G17510	UCH3	Ubiquitin C-terminal hydrolase 3 (UCH3);
AT4G17520	AT4G17520	RGG repeats nuclear RNA binding protein B;
AT4G17720	AT4G17720	RNA-binding (RRM/RBD/RNP motifs) family protein;
AT4G18100	AT4G18100	Ribosomal protein L32e;
AT4G19006	AT4G19006	26S proteasome non-ATPase regulatory subunit 13 homolog B;
AT4G19120	ERD3	S-adenosyl-L-methionine-dependent methyltransferases superfamily protein;
AT4G19710	AKHSDH2	Bifunctional aspartokinase/homoserine dehydrogenase 2, chloroplastic;
AT4G20850	TPP2	Tripeptidyl Peptidase II;
AT4G20980	AT4G20980	Eukaryotic translation initiation factor 3 subunit 7 (eIF-3);
AT4G21150	HAP6	Dolichyl-diphosphooligosaccharide--protein glycosyltransferase subunit 2;
AT4G22670	HIP1	FAM10 family protein At4g22670;
AT4G23460	AT4G23460	Beta-adaptin-like protein C;
AT4G24190	SHD	Chaperone protein htpG family protein;
AT4G25200	HSP23.6	Mitochondrion-localized small heat shock protein 23.6;
AT4G26910	AT4G26910	Dihydrolipoamide succinyltransferase;
AT4G26910	AT4G26910	Dihydrolipoamide succinyltransferase;
AT4G27090	AT4G27090	Ribosomal protein L14;
AT4G27130	AT4G27130	Translation initiation factor SUI1 family protein;
AT4G27270	AT4G27270	Probable NAD(P)H dehydrogenase (quinone) FQR1-like 1;

AT4G27640	AT4G27640	Uncharacterized protein At4g27640;
AT4G28300	AT4G28300	Protein of unknown function (DUF1421);
AT4G28390	AAC3	Mitochondrial ADP/ATP carrier protein;
AT4G29010	AIM1	Peroxisomal fatty acid beta-oxidation multifunctional protein AIM1;
AT4G29160	SNF7.1	Vacuolar protein sorting-associated protein 32 homolog 2;
AT4G29900	ACA10	Calcium-transporting ATPase 10, plasma membrane-type;
AT4G30330	AT4G30330	Small nuclear ribonucleoprotein family protein;
AT4G30440	GAE1	UDP-D-glucuronate 4-epimerase 1;
AT4G31080	AT4G31080	Integral membrane metal-binding family protein (DUF2296);
AT4G31300	PBA1	N-terminal nucleophile aminohydrolases (Ntn hydrolases) superfamily protein;
AT4G31480	AT4G31480	Coatomer subunit beta-1;
AT4G32910	AT4G32910	Nuclear pore complex protein NUP85;
AT4G33070	AT4G33070	Thiamine pyrophosphate dependent pyruvate decarboxylase family protein;
AT4G33090	APM1	Aminopeptidase M1;
AT4G33150	AT4G33150	Lysine-ketoglutarate reductase/bifunctional saccharopine dehydrogenase;
AT4G33250	EIF3K	Eukaryotic translation initiation factor 3 subunit K;
AT4G33640	AT4G33640	Costars family protein At4g33640;
AT4G34030	MCCB	Methylcrotonoyl-CoA carboxylase beta chain, mitochondrial;
AT4G34270	AT4G34270	TIP41-like family protein;
AT4G34450	AT4G34450	Coatomer gamma-2 subunit, putative/gamma-2 coat protein;
AT4G34640	SQS1	Squalene synthase;
AT4G34860	A/N-InvB	Plant neutral invertase family protein;
AT4G34890	XDH1	Xanthine dehydrogenase 1;
AT4G35220	AT4G35220	Cyclase family protein;
AT4G35790	PLDDELTA	Phospholipase D delta;
AT4G36910	LEJ2	CBS domain-containing protein CBSX1, chloroplastic;
AT4G37870	PCK1	Putative phosphoenolpyruvate carboxykinase (ATP-dependent)
AT4G37970	CAD6	Probable cinnamyl alcohol dehydrogenase 6;
AT4G37980	ELI3-1	Cinnamyl alcohol dehydrogenase 7;
AT4G37990	ELI3-2	Cinnamyl alcohol dehydrogenase 8;
AT4G38460	GGR	Geranylgeranyl pyrophosphate synthase small subunit, chloroplastic;
AT4G38580	FP6	Heavy metal-associated isoprenylated plant protein 26;

AT4G38600	KAK	HEAT repeat - HECT-domain (ubiquitin-transferase);
AT4G38710	AT4G38710	Eukaryotic translation initiation factor 4B3;
AT4G39080	VHA-A3	V-type proton ATPase subunit a3;
AT4G39230	AT4G39230	NmrA-like negative transcriptional regulator family protein;
AT4G39260	GRP8	Cold circadian rhythm and RNA binding protein 1;
AT5G01320	AT5G01320	Thiamine pyrophosphate dependent pyruvate decarboxylase family protein;
AT5G01600	FER1	Ferritin-1, chloroplastic;
AT5G02500	HSC70-1	Probable mediator of RNA polymerase II transcription subunit 37e;
AT5G02560	HTA12	Encodes HTA12, a histone H2A protein
AT5G02790	GSTL3	Glutathione S-transferase family protein;
AT5G04800	AT5G04800	Ribosomal S17 family protein;
AT5G06130	AT5G06130	Protein ORANGE-LIKE, chloroplastic;
AT5G06460	UBA2	Ubiquitin-activating enzyme E1 2;
AT5G06970	AT5G06970	Protein of unknown function (DUF810);
AT5G07350	Tudor1	Ribonuclease TUDOR 1;
AT5G08100	ASPGA1	N-terminal nucleophile aminohydrolases (Ntn hydrolases) superfamily protein;
AT5G08290	YLS8	mRNA splicing factor, thioredoxin-like U5 snRNP;
AT5G08530	CI51	NADH dehydrogenase [ubiquinone] flavoprotein 1, mitochondrial;
AT5G08570	AT5G08570	Pyruvate kinase family protein;
AT5G09500	AT5G09500	Ribosomal protein S19 family protein;
AT5G09590	MTHSC70-2	Heat shock 70 kDa protein 10, mitochondrial;
AT5G09650	PPa6	Soluble inorganic pyrophosphatase 6, chloroplastic;
AT5G10770	AT5G10770	Eukaryotic aspartyl protease family protein;
AT5G10840	EMP1	Endomembrane protein 70 protein family;
AT5G11040	TRS120	Trafficking protein particle complex II-specific subunit 120 homolog;
AT5G11560	AT5G11560	Catalytics mRNA;
AT5G11710	AT5G11710	Clathrin interactor EPSIN 1;
AT5G12020	HSP17.6II	17.6 kDa class II heat shock protein (HSP17.6II);
AT5G12110	AT5G12110	Glutathione S-transferase, C-terminal-like;
AT5G12470	AT5G12470	Protein reticulata-related 4, chloroplastic;
AT5G13440	AT5G13440	Cytochrome b-c1 complex subunit Rieske-2, mitochondrial;
AT5G13450	ATP5	ATP synthase subunit O, mitochondrial;

AT5G13490	AAC2	ADP,ATP carrier protein 2, mitochondrial;
AT5G13850	NACA3	Nascent polypeptide-associated complex subunit alpha-like protein 3;
AT5G13930	TT4	Chalcone and stilbene synthase family protein;
AT5G14780	FDH	Formate dehydrogenase, chloroplastic/mitochondrial;
AT5G14930	SAG101	Senescence-associated carboxylesterase 101;
AT5G15270	AT5G15270	RNA-binding KH domain-containing protein;
AT5G16970	AER	NADPH-dependent oxidoreductase 2-alkenal reductase;
AT5G17020	XPO1A	Protein exportin 1A;
AT5G17380	AT5G17380	Thiamine pyrophosphate dependent pyruvate decarboxylase family protein;
AT5G17710	EMB1241	Co-chaperone GrpE family protein;
AT5G17770	CBR	NADH-cytochrome b5 reductase 1;
AT5G17920	ATMS1	5-Methyltetrahydropteroyltriglutamate--homocysteine methyltransferase 1;
AT5G19440	AT5G19440	NAD(P)-binding Rossmann-fold superfamily protein;
AT5G19820	emb2734	Uncharacterized protein At5g19820;
AT5G20160	AT5G20160	Ribosomal protein L7Ae/L30e/S12e/Gadd45 family protein;
AT5G20280	SPS1F	Sucrose phosphate synthase 1F;
AT5G20490	XIK	Myosin family protein with Dil domain;
AT5G20720	CPN20	20 kDa chaperonin, chloroplastic;
AT5G20890	AT5G20890	TCP-1/cpn60 chaperonin family protein;
AT5G22470	AT5G22470	NAD+ ADP-ribosyltransferases;
AT5G22780	AT5G22780	Adaptor protein complex AP-2, alpha subunit;
AT5G23190	CYP86B1	Cytochrome P450, family 86, subfamily B, polypeptide 1;
AT5G23540	AT5G23540	26S proteasome non-ATPase regulatory subunit 14 homolog;
AT5G24400	EMB2024	Probable 6-phosphogluconolactonase 5, chloroplastic;
AT5G25260	AT5G25260	SPFH/Band 7/PHB domain-containing membrane-associated protein;
AT5G25450	AT5G25450	Cytochrome bd ubiquinol oxidase, 14kDa subunit;
AT5G25757	AT5G25757	Eukaryotic translation initiation factor 3 subunit L;
AT5G25880	NADP-ME3	Malic enzyme encoded by NADP-ME3 gene;
AT5G26710	AT5G26710	Glutamyl/glutaminyl-tRNA synthetase, class Ic;
AT5G26830	AT5G26830	Threonine-tRNA ligase, mitochondrial 1;
AT5G27030	F2P16.14	Topless-related protein 3;
AT5G27120	AT5G27120	NOP56-like pre RNA processing ribonucleoprotein;

AT5G28060	AT5G28060	Ribosomal protein S24e family protein;
AT5G28540	BIP1	Mediator of RNA polymerase II transcription subunit 37a;
AT5G28830	AT5G28830	Calcium-binding EF hand family protein;
AT5G34850	PAP26	Bifunctional purple acid phosphatase 26;
AT5G35360	CAC2	Acetyl Co-enzyme A carboxylase biotin carboxylase subunit;
AT5G35700	FIM5	Fimbrin-like protein 2;
AT5G36110	CYP716A1	Cytochrome P450, family 716, subfamily A, polypeptide 1;
AT5G36210	AT5G36210	Alpha/beta-hydrolase superfamily protein;
AT5G37150	AT5G37150	P-loop containing nucleoside triphosphate hydrolases superfamily protein;
AT5G37600	GLN1-1	Glutamine synthetase cytosolic isozyme 1-1;
AT5G37770	TCH2	EF-hand calcium-binding protein family;
AT5G37780	CAM1	Calmodulin involved in thigmomorphogenesis;
AT5G39410	AT5G39410	Mitochondrial saccharopine dehydrogenase-like oxidoreductase At5g39410;
AT5G40770	PHB3	Prohibitin-3, mitochondrial;
AT5G42190	ASK2	E3 ubiquitin ligase SCF complex subunit SKP1/ASK1 family protein;
AT5G42420	AT5G42420	Nucleotide-sugar transporter family protein;
AT5G43940	HOT5	GroES-like zinc-binding dehydrogenase family protein;
AT5G45160	RL2	Root hair defective 3 GTP-binding protein (RHD3);
AT5G46070	AT5G46070	Guanylate-binding family protein;
AT5G46280	MCM3	Minichromosome maintenance (MCM2/3/5) family protein;
AT5G46860	VAM3	Syntaxin/t-SNARE family protein;
AT5G47540	AT5G47540	Putative MO25-like protein At5g47540;
AT5G48370	AT5G48370	Thioesterase/thiol ester dehydrase-isomerase superfamily protein;
AT5G49360	BXL1	Beta-D-xylosidase 1;
AT5G49460	ACLB-2	ATP-citrate synthase beta chain protein 2;
AT5G51970	AT5G51970	GroES-like zinc-binding alcohol dehydrogenase family protein;
AT5G52200	I-2	Phosphoprotein phosphatase inhibitor;
AT5G53330	AT5G53330	Ubiquitin-associated/translation elongation factor EF1B protein;
AT5G53400	BOB1	HSP20-like chaperones superfamily protein;
AT5G53480	AT5G53480	ARM repeat superfamily protein;
AT5G54310	AGD5	Probable ADP-ribosylation factor GTPase-activating protein AGD5;
AT5G54500	FQR1	Auxin-dependent flavin mononucleotide-binding quinone reductase;

AT5G54960	PDC2	Pyruvate decarboxylase-2;
AT5G55160	SUMO2	Small ubiquitin-like modifier (SUMO) polypeptide;
AT5G55200	MGE1	GrpE protein homolog 1, mitochondrial;
AT5G55240	ATPXG2	Arabidopsis thaliana peroxygenase 2;
AT5G55940	emb2731	ER membrane protein complex subunit 8/9 homolog;
AT5G56680	SYNC1	Class II aminoacyl-tRNA and biotin synthetases superfamily protein;
AT5G56710	AT5G56710	Ribosomal protein L31e family protein;
AT5G58070	TIL	Temperature-induced lipocalin-1;
AT5G59950	AT5G59950	RNA-binding (RRM/RBD/RNP motifs) family protein;
AT5G59970	At1g07660	Histone superfamily protein;
AT5G60640	PDIL1-4	Protein disulfide isomerase-like (PDIL) protein 1-4;
AT5G60860	RABA1f	Ras-related protein RABA1f;
AT5G60980	AT5G60980	Nuclear transport factor 2 with RNA binding (RRM-RBD-RNP motifs) domain;
AT5G61170	AT5G61170	Ribosomal protein S19e family protein;
AT5G62350	AT5G62350	Plant invertase/pectin methylesterase inhibitor superfamily protein;
AT5G62670	HA11	ATPase 11, plasma membrane-type;
AT5G62890	AT5G62890	Xanthine/uracil permease family protein;
AT5G63620	AT5G63620	GroES-like zinc-binding alcohol dehydrogenase family protein;
AT5G64130	AT5G64130	cAMP-regulated phosphoprotein 19-related protein;
AT5G64250	AT5G64250	2-Nitropropane dioxygenase-like protein;
AT5G65750	AT5G65750	2-Oxoglutarate dehydrogenase, E1 component;
AT5G66030	ATGRIP	Protein involved in Golgi protein trafficking;
AT5G67500	VDAC2	Mitochondrial outer membrane protein porin 2;
ATCG00480	PB	ATP synthase subunit beta, chloroplastic;
ATCG00490	RBCL	Ribulose bisphosphate carboxylase large chain;
ATMG00665	NAD5B	NADH-ubiquinone oxidoreductase chain 5.

Supplementary Figure S1.

Supplementary Figure S2.

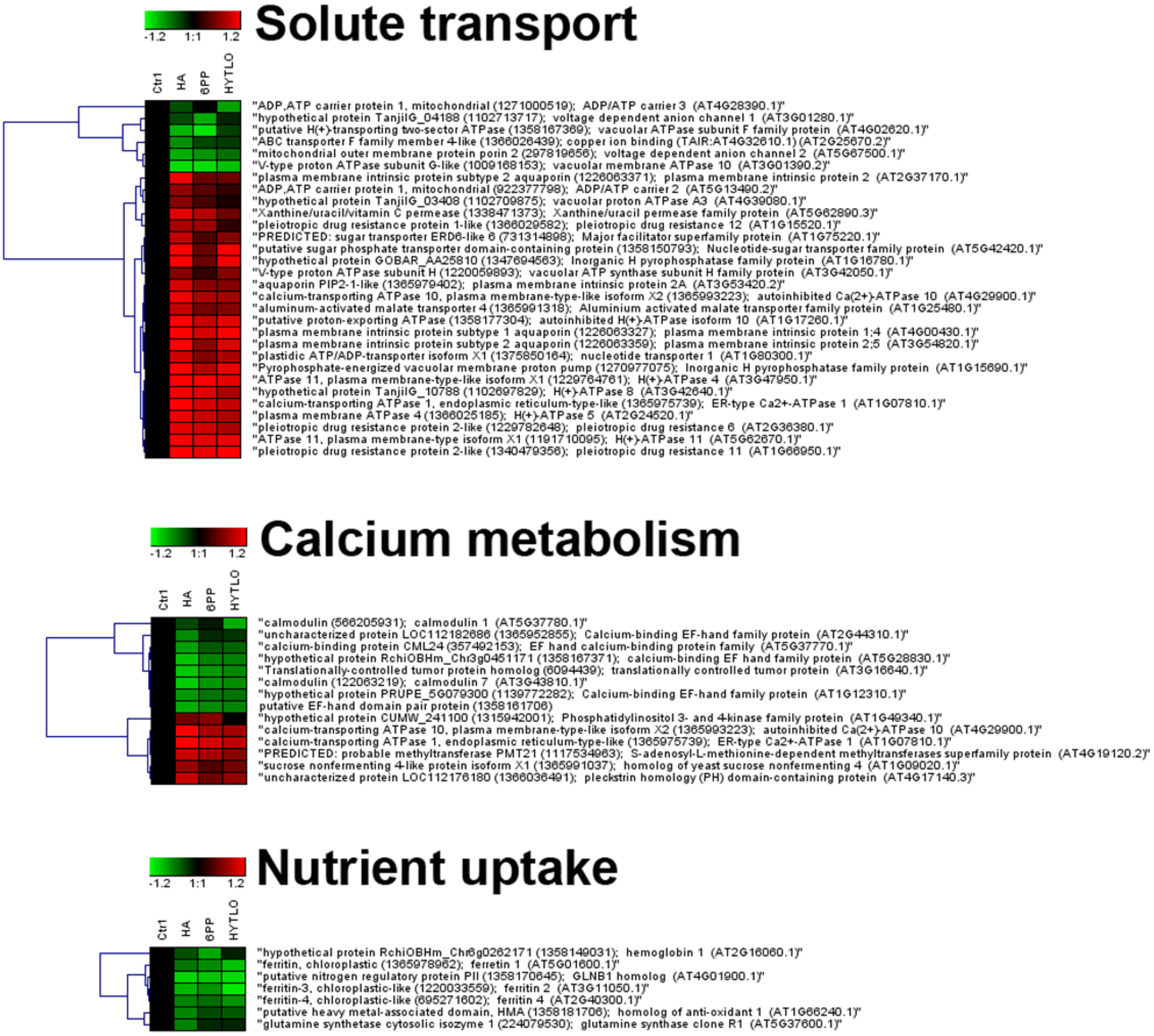

Supplementary Figure S3.

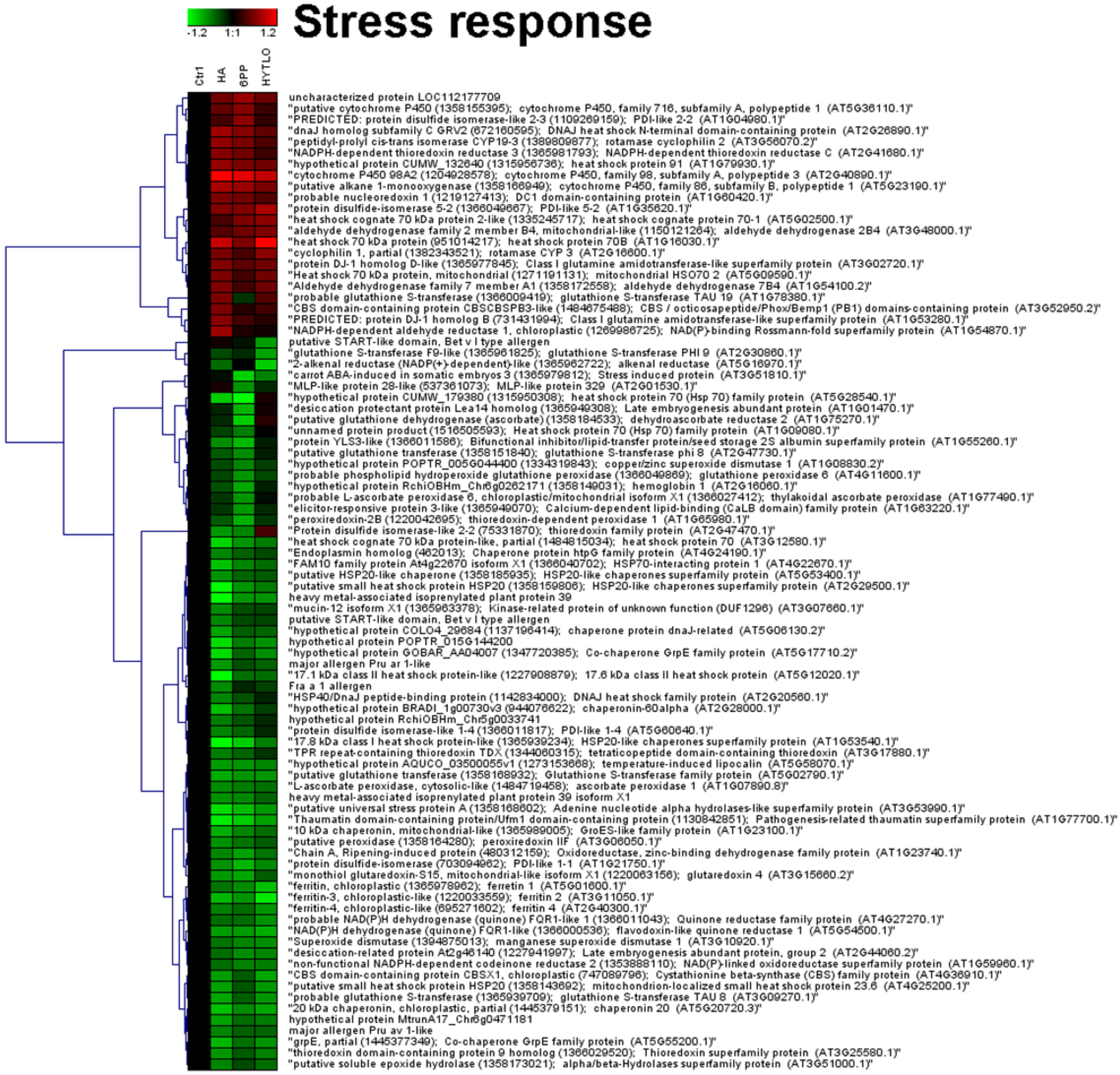

Supplementary Figure S4.

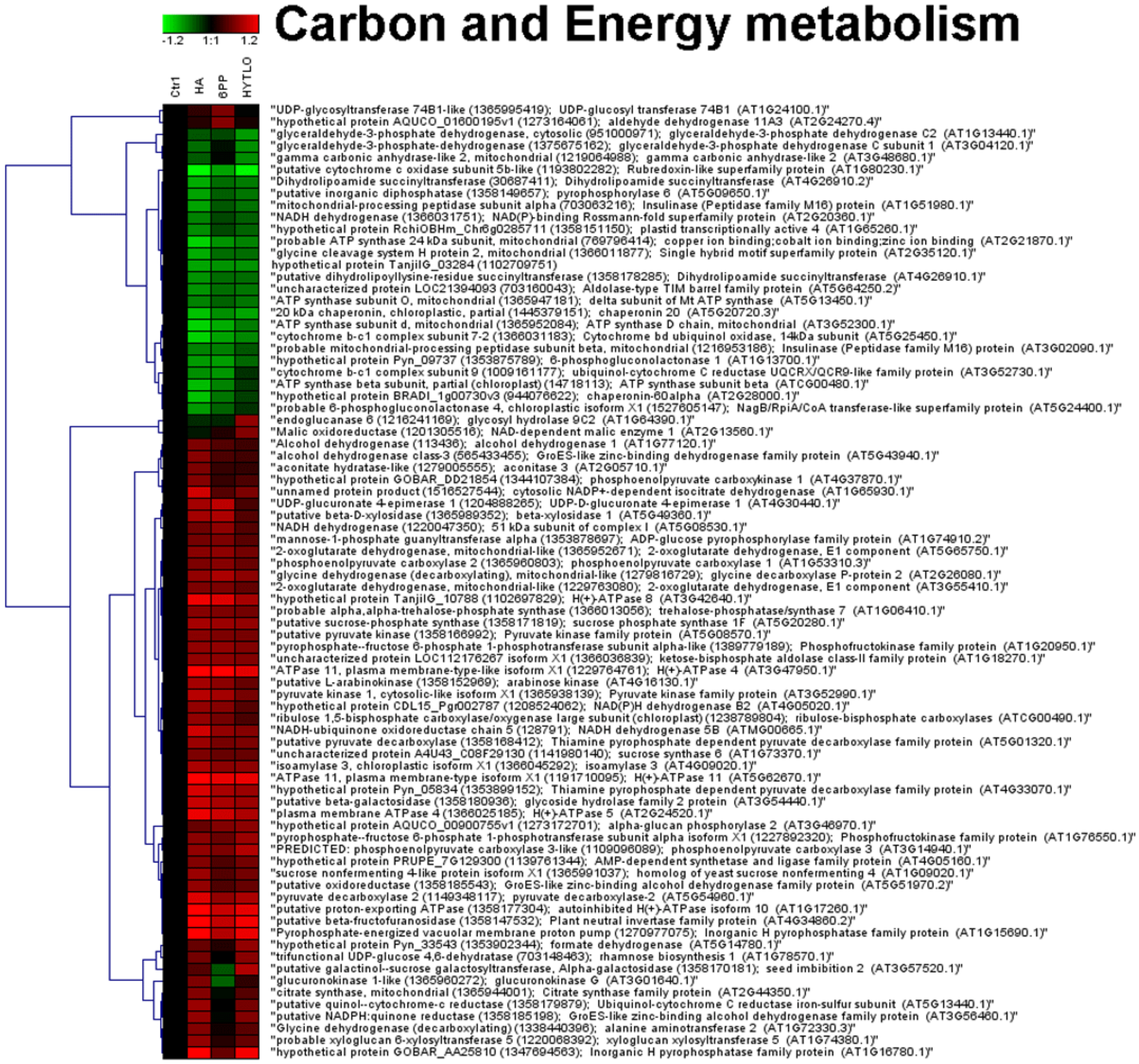

Supplementary Figure S5.

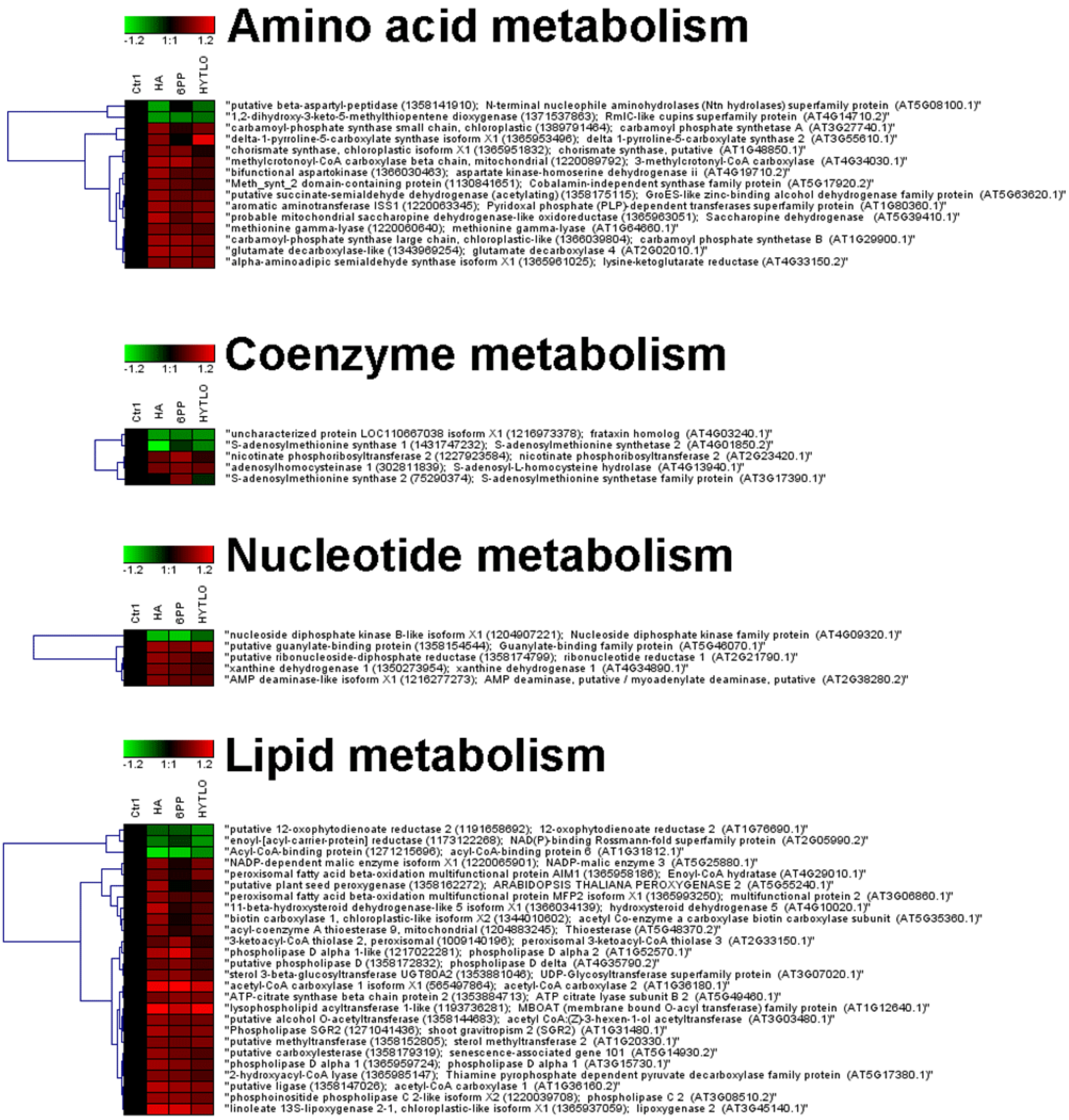

Supplementary Figure S6.

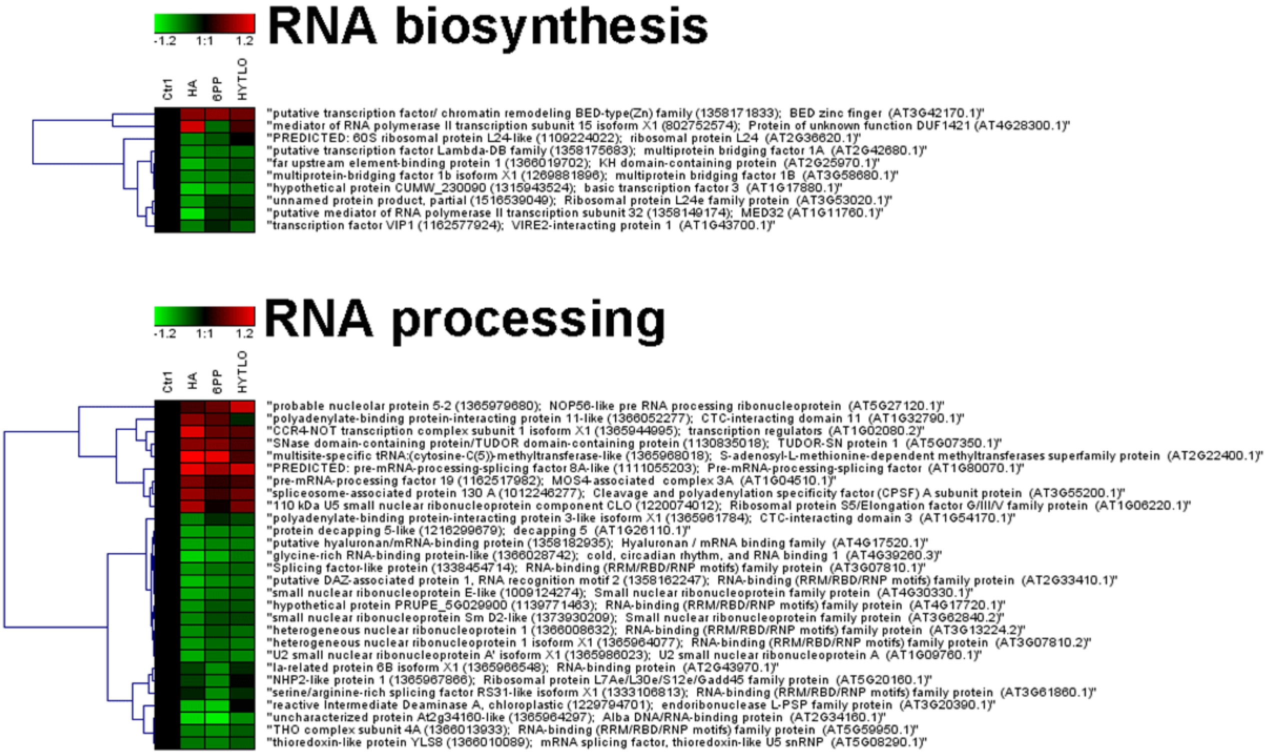

Supplementary Figure S7.

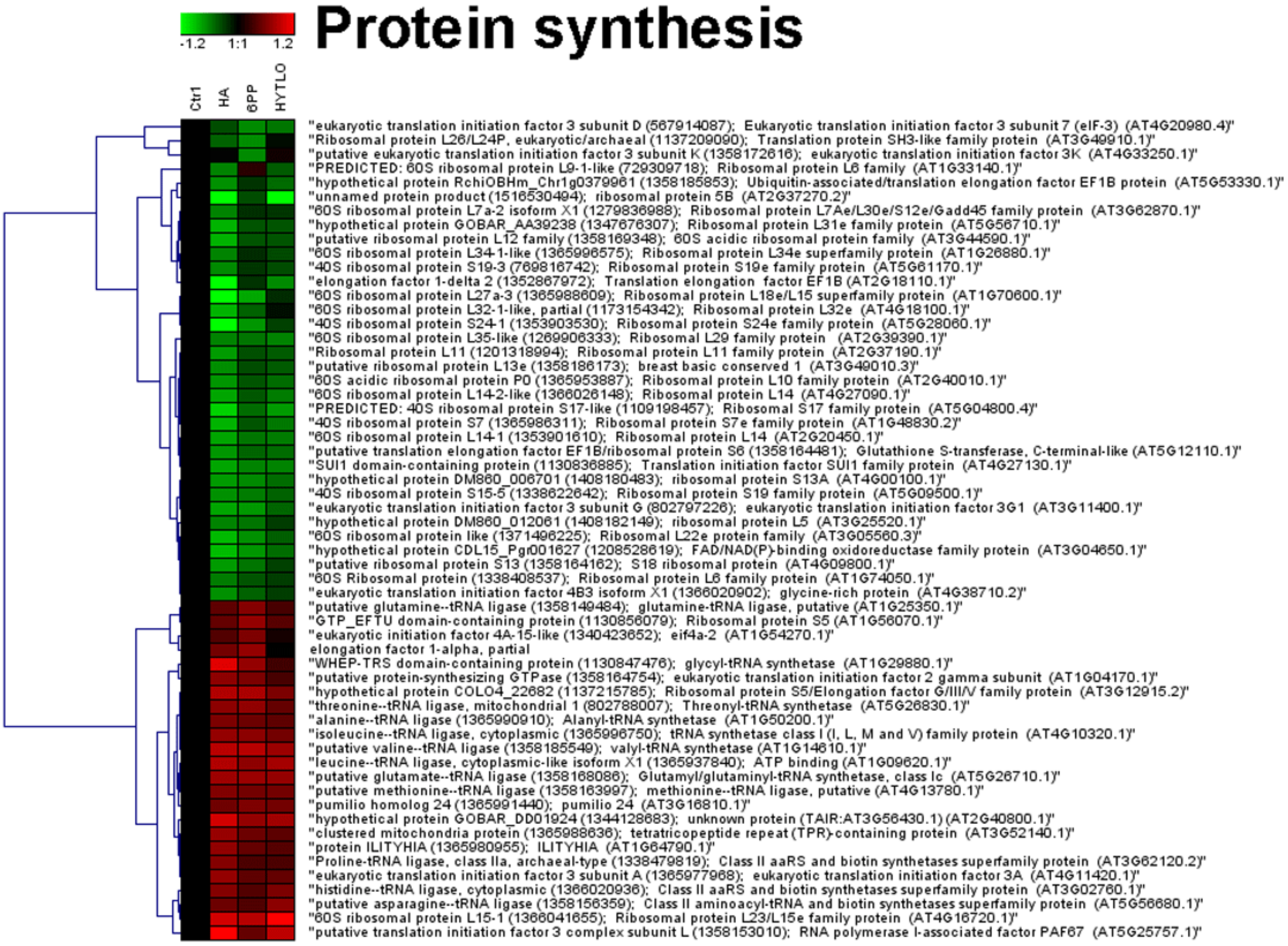

Supplementary Figure S8.

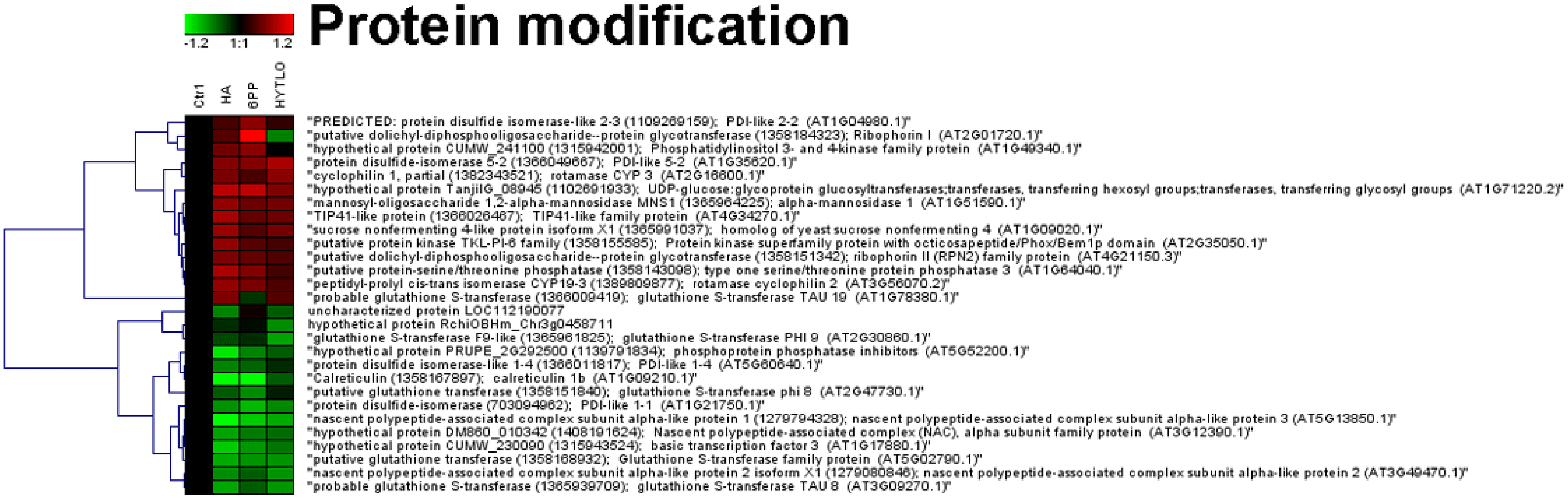

Supplementary Figure S9.

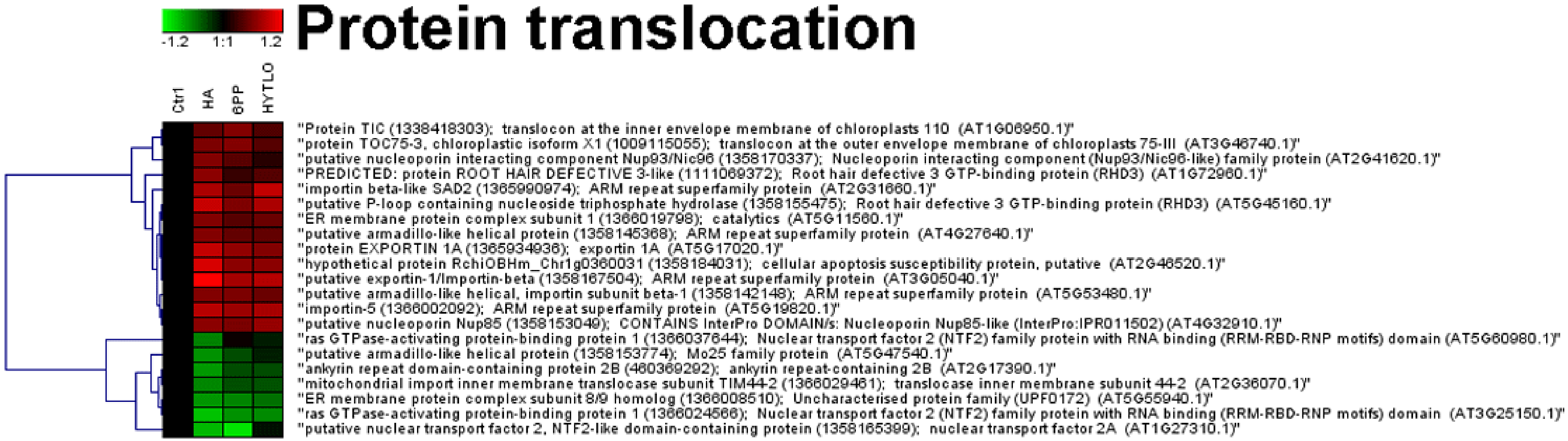

Supplementary Figure S10.

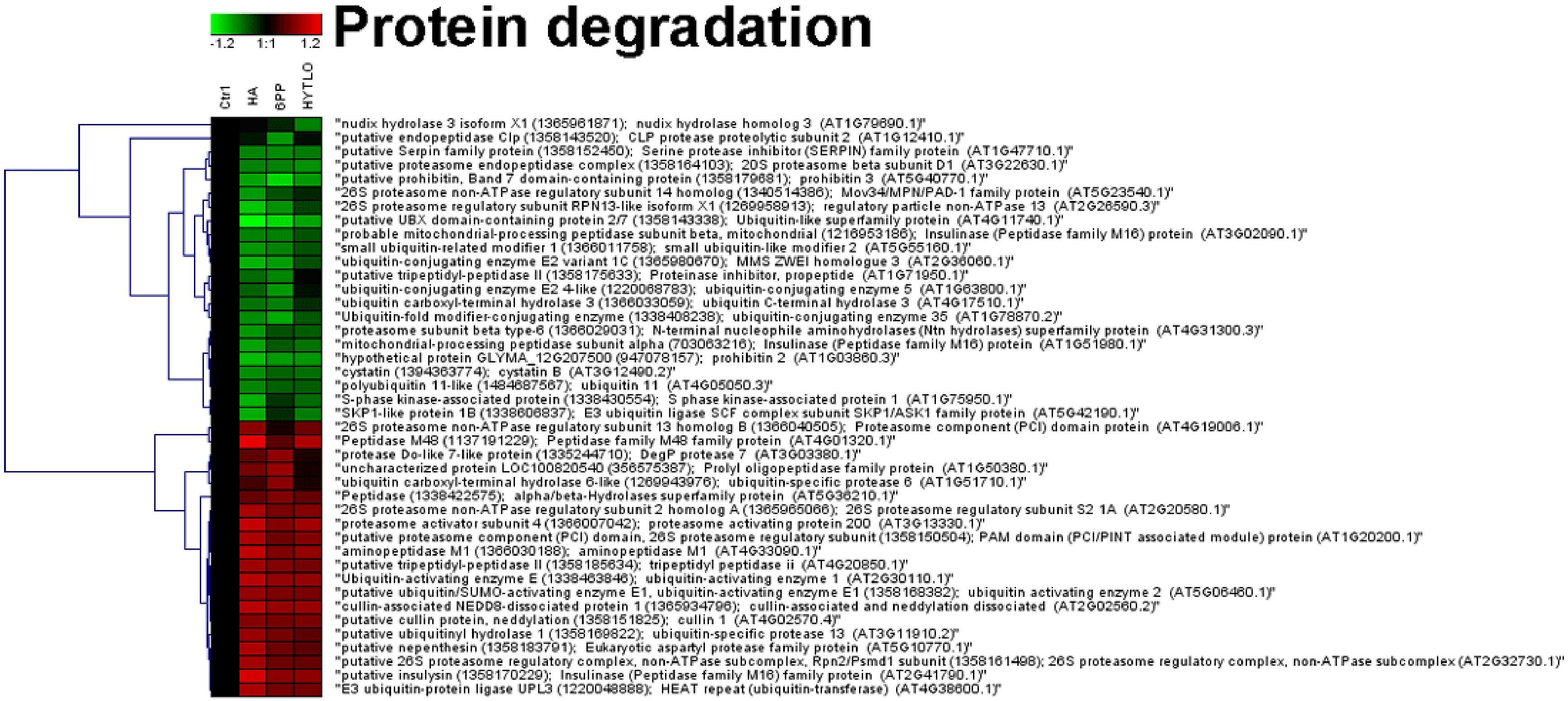

Supplementary Figure S11.

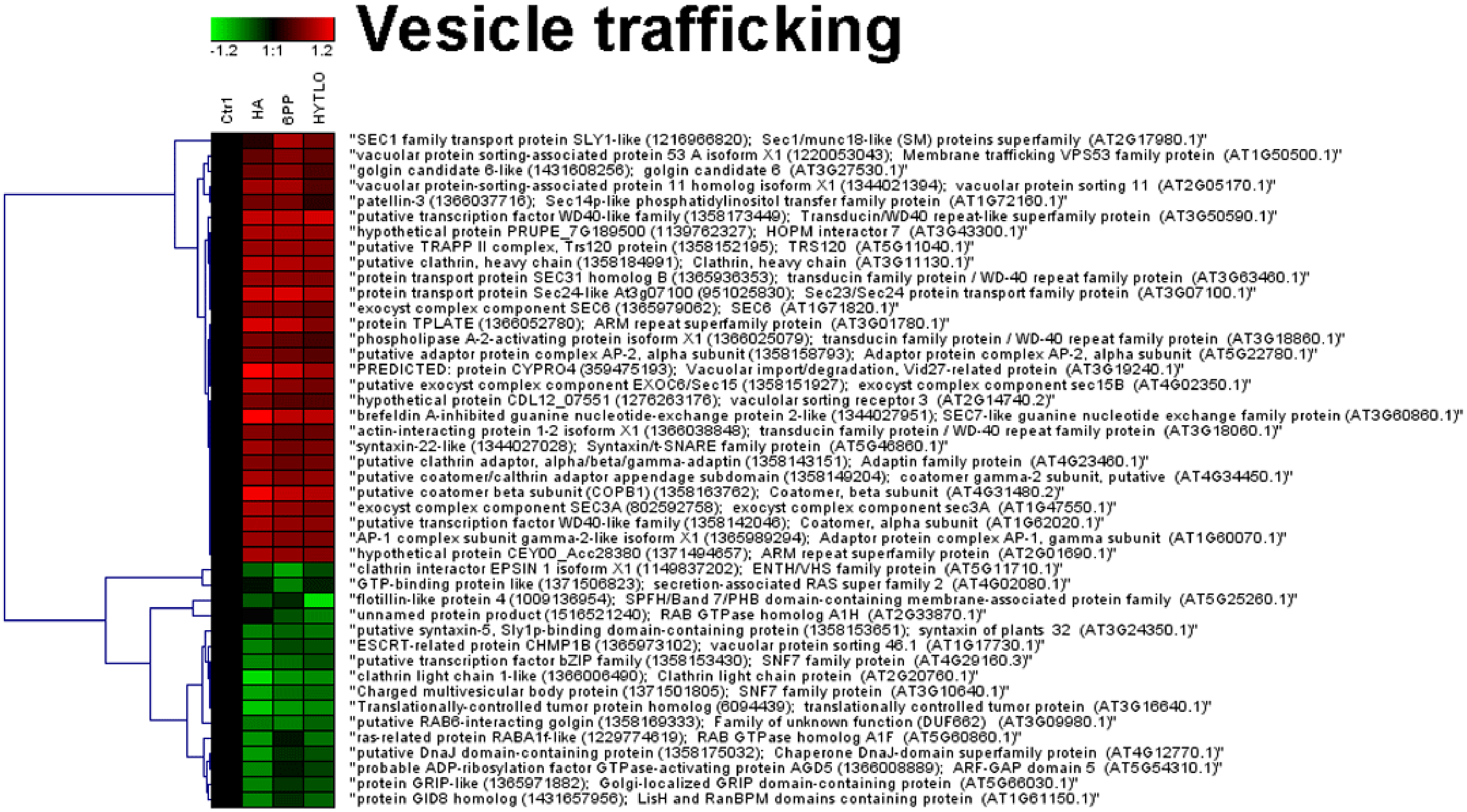

Supplementary Figure S12.

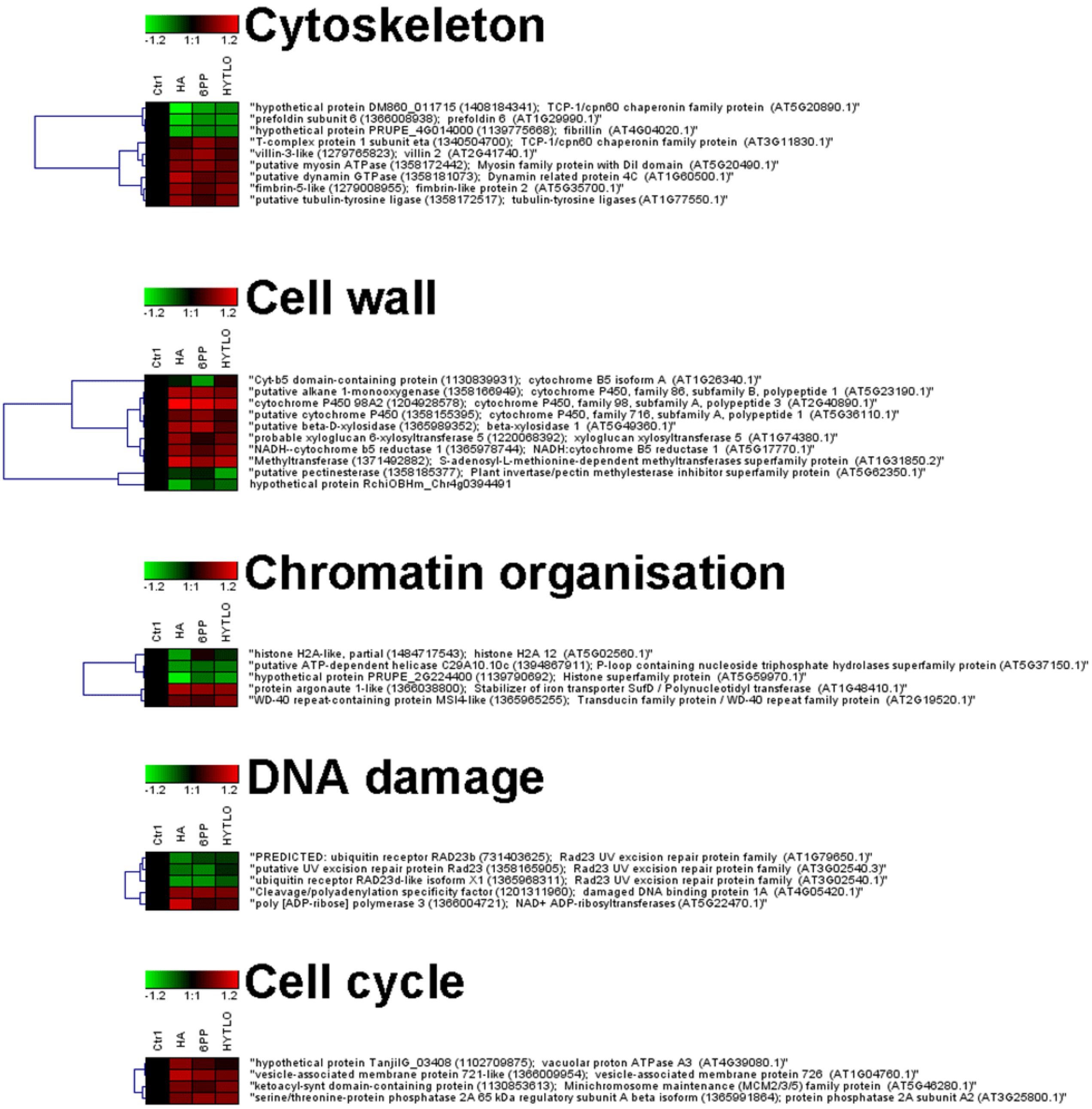

Supplementary Figure S13.

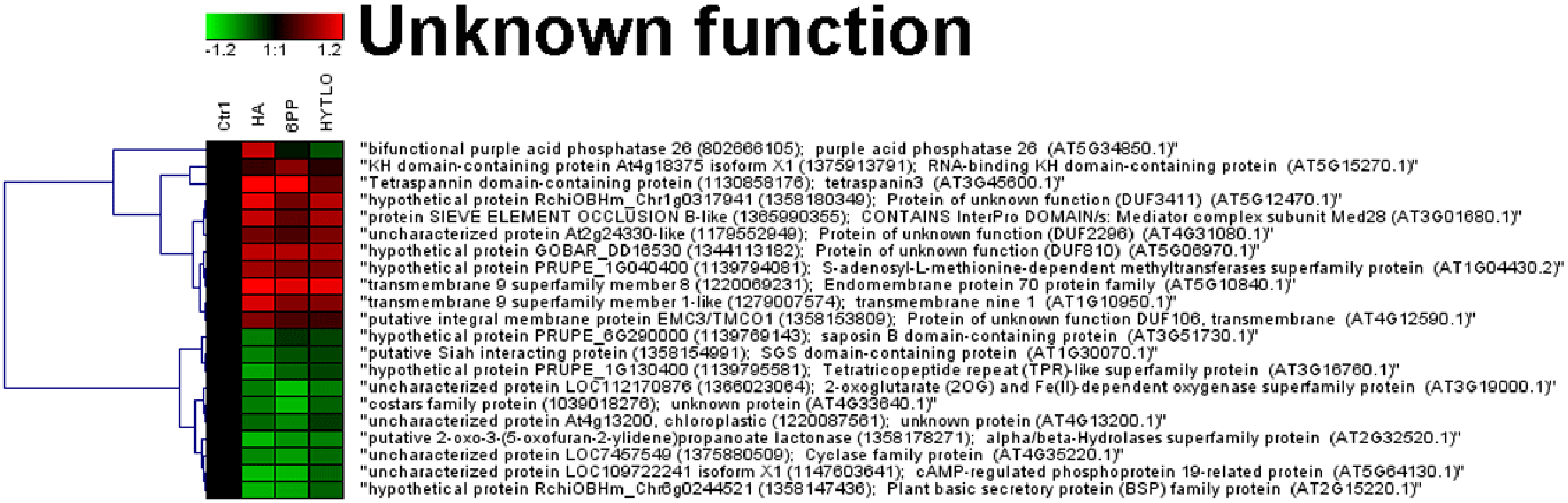

Supplement: Supplementary file 1 — jf0c01438_si_001.pdf [file jf0c01438_si_001.pdf]
